# Ex vivo pretreatment with venetoclax boosts antileukemic efficacy of therapeutic **γδ** T cells in AML models

**DOI:** 10.1172/JCI202932

**Published:** 2026-07-23

**Authors:** Xingchi Chen, Lin Zhang, Bingbing Yan, Yinqiang Sui, Weiwei Ma, Hui Zhao, Yining Wang, Kepeng Yang, Jiewen Ma, Baolin Tang, Yonghui Zhang, Xiaoyu Zhu

**Affiliations:** 1 Department of Hematology, Centre for Leading Medicine and Advanced Technologies of IHM, the First Affiliated Hospital of USTC, Division of Life Sciences and Medicine, and; 2 Blood and Cell Therapy Institute, Division of Life Sciences and Medicine, University of Science and Technology of China (USTC), Hefei, China.; 3 Anhui Provincial Key Laboratory of Blood Research and Applications, Hefei, China.; 4 Tsinghua-Peking Center for Life Sciences, School of Pharmaceutical Sciences, Tsinghua University, Beijing, China.; 5 UnicetBio Co., Ltd., Beijing, China.

**Keywords:** Hematology, Immunology, Cancer immunotherapy, Immunotherapy, Leukemias

## Abstract

Ex vivo engineering strategies for adoptive αβ T cell therapies increasingly use pharmacological modulation to improve survival, expansion, and antitumor activity. Short-term exposure to the B cell lymphoma-2 (BCL-2) inhibitor venetoclax during αβ T cell manufacturing enhances apoptotic priming and effector persistence, suggesting a route to strengthen other T cell lineages. γδ T cells share cytotoxic properties with αβ T cells but recognize targets independently of major histocompatibility complex and show low alloreactivity, supporting off-the-shelf use in acute myeloid leukemia (AML). Whether such conditioning benefits γδ T cells was unknown. Here, we show that ex vivo venetoclax pretreatment enhances the antileukemic efficacy of therapeutic γδ T cells and chimeric antigen receptor (CAR) γδ T cells. Venetoclax-pretreated γδ T cells displayed increased cytotoxicity and proliferation with reduced exhaustion, yielding superior control of AML blasts and xenografts. These functional gains coincided with elevated mitochondrial content and a fatty acid oxidation metabolic profile. In vivo, venetoclax-pretreated γδ T cells achieved durable disease suppression, and the same conditioning improved CAR γδ T cell efficacy. Together, these results show that short-term BCL-2 inhibition enhances γδ T cell cytotoxicity and persistence. Incorporating venetoclax pretreatment into γδ T cell manufacturing may improve therapeutic efficacy and inform next-generation γδ T cell therapies for AML.

## Abstract 2

A brief venetoclax treatment strengthens γδ T-cell therapies against acute myeloid leukemia by improving cell fitness, tumor killing, and survival in mice.

## Graphical abstract

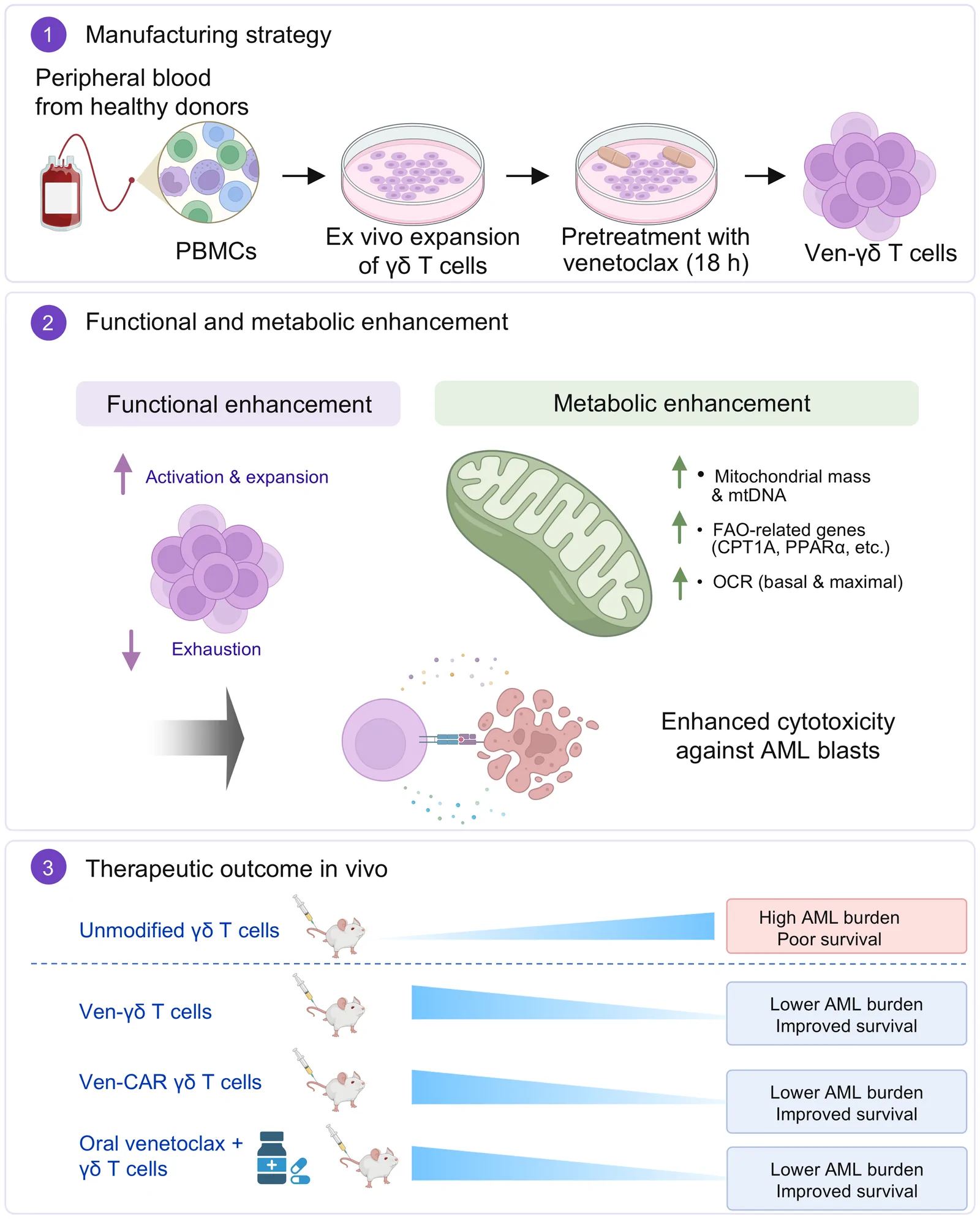

## Introduction

Acute myeloid leukemia (AML) is an aggressive hematologic malignancy that often emerges from clonal hematopoiesis driven by genetic and environmental risks, with clonal expansion of poorly differentiated myeloid progenitors, leading to bone marrow (BM) failure and systemic disease (1). Despite progress in chemotherapeutic and molecularly targeted approaches, outcomes remain poor for many patients, particularly in relapsed or refractory settings (2). Recent advances in cell-based therapies, including engineered T cell and NK cell strategies, have demonstrated promising antileukemic activity in early-phase trials, raising the potential for durable responses and expanding treatment options for AML (3, 4).

The efficacy and persistence of chimeric antigen receptor (CAR) T and T cell receptor (TCR) engineered T cell therapies are shaped by the metabolic and apoptotic landscapes of engineered T cells (5). Increasingly, engineering pipelines for generating therapeutic cells to treat hematologic malignancies are incorporating metabolic and apoptotic reprogramming strategies to enhance function and durability (5, 6). Pharmacological tools such as venetoclax, a B cell lymphoma-2 (BCL-2) inhibitor, have shown potential in selectively modulating apoptotic priming to improve T cell survival and antitumor activity (7–9). In parallel, inhibitors targeting phosphatidylinositol-3-kinase (PI3K), protein kinase B (AKT), and isocitrate dehydrogenase (IDH2) are being explored to reshape T cell differentiation, exhaustion profiles, and metabolic fitness (10–13). These interventions to rewire T cell fate and functionality represent a growing toolbox for overcoming limitations of adoptive therapies in hematologic malignancies.

While metabolic and apoptotic reprogramming strategies have been adopted to improve αβ T cell–based therapies, including CAR T and TCR T products, another T cell lineage — γδ T cells — has distinct biology and is gaining momentum in clinical translation. Unlike αβ T cells, γδ T cells recognize stress ligands in an MHC-independent manner and possess innate-like cytotoxicity, tissue tropism, and low risk of graft-versus-host disease, making them attractive for off-the-shelf applications (14, 15). However, whether engineered or expanded γδ T cells can similarly benefit from metabolic or apoptotic preconditioning strategies remains unexplored. As γδ T cell products enter clinical trials (16–18), understanding whether these distinct cells respond to such interventions in desirable ways can inform the design of future immunotherapies.

In this study, we investigated whether pharmacological modulation with the BCL-2 inhibitor venetoclax enhances the functional properties of therapeutic γδ T cells in the context of treating AML. Reanalysis of a published single-cell RNA-sequencing (scRNA-seq) dataset showed that patients with relapsed AML had reduced γδ T cell abundance and transcriptional profiles consistent with impaired cytotoxicity and disrupted mitochondrial metabolism. Guided by these findings, we tested whether ex vivo venetoclax pretreatment enhances γδ T cell function. Venetoclax-pretreated γδ T cells displayed increased cytotoxic capacity, proliferation, and persistence with reduced exhaustion, resulting in stronger antileukemic activity in vitro and in AML xenograft models. Profiling revealed increased mitochondrial mass and respiration, along with a fatty acid oxidation (FAO)–oxidative phosphorylation (OXPHOS)–linked metabolic shift associated with the improved functional profile. Finally, venetoclax pretreatment also enhanced the in vivo efficacy of C-type lectin-like molecule-1–directed (CLL-1–directed) CAR γδ T cells, indicating a broadly applicable strategy to improve γδ T cell–based immunotherapies for AML.

## Results

### Reanalysis of clinical scRNA-seq data reveals γδ T cell deficiency in relapsed patients with AML.

The genesis of our present study was a reanalysis of a publicly available scRNA-seq dataset from Mathioudaki et al. (19) that examined a cohort of patients with AML who received an allogeneic hematopoietic stem cell transplantation (allo-HSCT) therapy. Among other analyses, the original study evaluated the cohort by splitting patients into those who achieved complete remission (CR) versus those who experienced relapse, and a striking finding was their discovery that the frequencies of both αβ T cells (CD8^+^) and γδ T cells (TRDC^+^, TRGC1^+^) were markedly higher in the CR patient group compared with the relapsed patients. This association between higher posttransplant γδ T cell abundance and improved clinical outcomes has also been reported in earlier allo-HSCT cohorts (20–24).

Our previous studies have examined the basic biology of γδ T cells (25–27). In light of this interest, we harnessed the scRNA-seq dataset for a focused analysis that extended beyond the scope of the original Mathioudaki et al. study. γδ T cells are circulating immune cells that undergo an activation process through which they sense and respond to “danger signals” indicating infection, stress, or cancer, notably doing so in an MHC-independent manner, thus distinguishing them from αβ T cells, which require antigens to be presented by MHC molecules (14). Once activated, γδ T cells quickly proliferate, produce inflammatory cytokines, and develop a potent cytotoxic response to kill a target cell (14).

**Figure 1:**
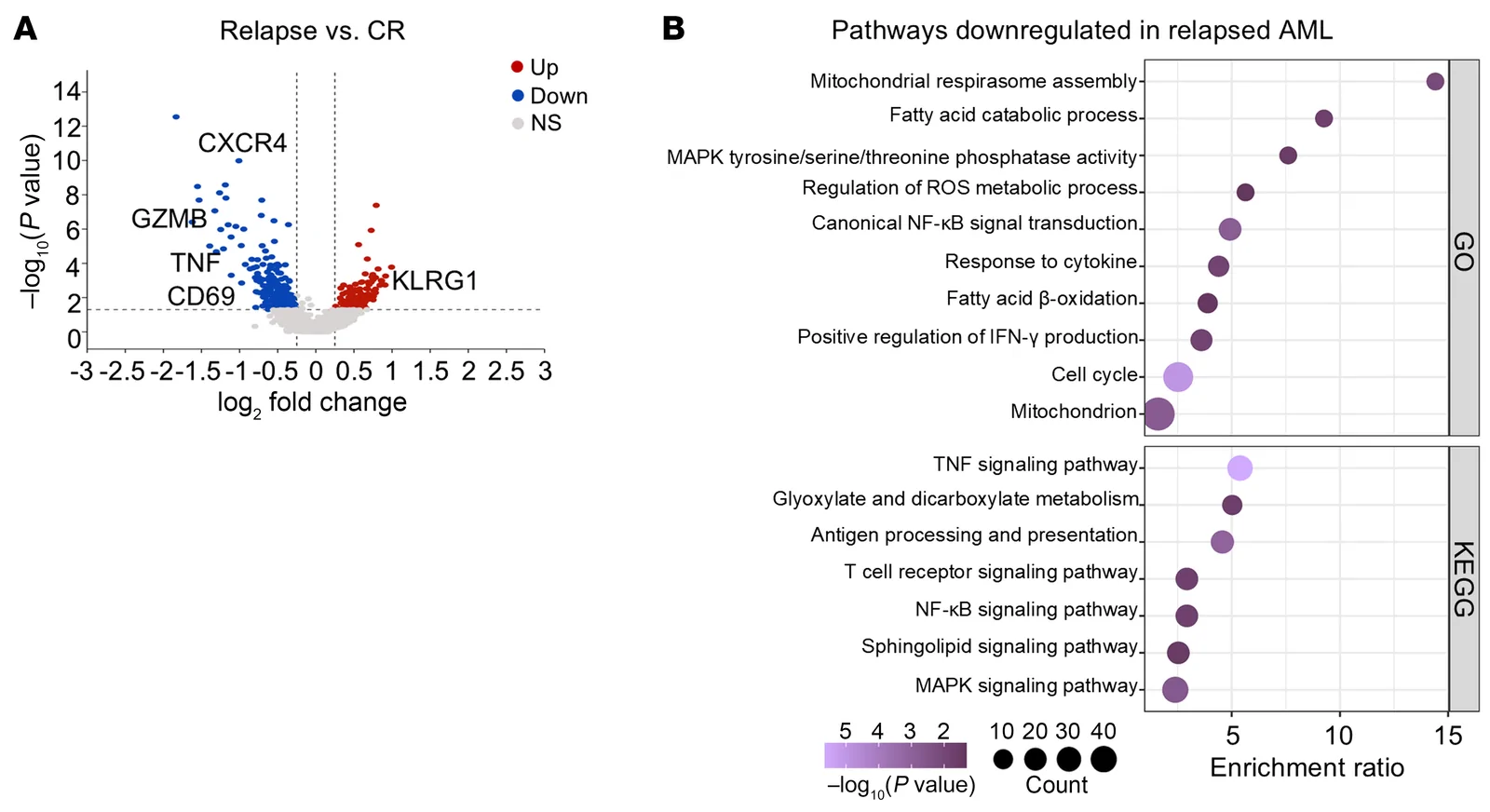
Reanalysis of clinical scRNA-seq data reveals functional impairment and metabolic dysregulation of BM γδ T cells in relapsed AML after allo-HSCT. (**A**) Volcano plot of differentially expressed genes in BM γδ T cells from relapsed AML patients compared with those in CR after HSCT. Red and blue dots indicate significantly upregulated and downregulated genes, respectively (*P* < 0.05, log_2_FC > 0.25 or < –0.25). FC, fold-change. (**B**) Representative Gene Ontology (GO) terms and Kyoto Encyclopedia of Genes and Genomes (KEGG) pathways enriched among genes downregulated in relapsed AML.

Our focused reanalysis examined a total of 294 γδ T cells, with an average of 1,355 high-confidence genes per cell. Regarding specific genes, the γδ T cells of patients with relapsed AML showed markedly reduced expression levels for cytotoxic and activation-associated genes, including granzyme B (GZMB), CD69, TNF, and CXCR4. Conversely, the level of the senescence marker killer cell lectin like receptor subfamily G member 1 (KLRG1) was substantially higher in the γδ T cells of the relapsed patient group (Supplemental Table 1; supplemental material available online with this article; https://doi.org/10.1172/JCI202932DS1). These signals collectively support that the γδ T cells of the relapsed AML patient group exhibit impaired immune effector function (Figure 1A).

Gene Ontology (GO) and Kyoto Encyclopedia of Genes and Genomes (KEGG) enrichment analysis indicated potentially informative changes in terms related to metabolism: the relapsed patient γδ T cells showed reductions for the terms fatty-acid catabolic process, fatty acid beta-oxidation, and sphingolipid signaling pathway. There were also a large variety of terms related to mitochondria identified in the GO analysis (e.g., mitochondrion, mitochondrial respirasome assembly, etc.) (Figure 1B and Supplemental Tables 2 and 3). Thus, patients with relapsed AML have a reduced overall number of γδ T cells compared with patients who achieve CR, and the γδ T cells of the relapsed patients have transcriptomes consistent with impaired immune effector function and dysregulation of metabolism and mitochondria.

### An ex vivo pretreatment with venetoclax boosts the cytotoxic capacity of γδ T cells against AML.

Given the better clinical outcomes of the CR versus relapsed AML patients, and considering the reduced numbers and apparently impaired functions of γδ T cells in patients with relapsed AML, we inferred that γδ T cells may somehow promote an antileukemic effect (22, 24, 28–30). A variety of recent studies have explored use of an ex vivo pretreatment for therapeutic T cells using the BCL-2 inhibitor venetoclax. These studies have collectively reported that the venetoclax pretreatment boosted the antileukemic efficacy for αβ T cells (8, 31) and CAR T cells (9) and have reported specific phenotypes for venetoclax-pretreated αβ T cells, including improvements in expansion, immune activation, and cytotoxic capacity, as well as memory-like phenotypes. Here, pursuing the potential of therapeutic γδ T cells for treating AML, we tested whether ex vivo exposure of γδ T cells to venetoclax confers similar beneficial outcomes as reported for αβ T cells, starting with boosted cytotoxic capacity.

**Figure 2:**
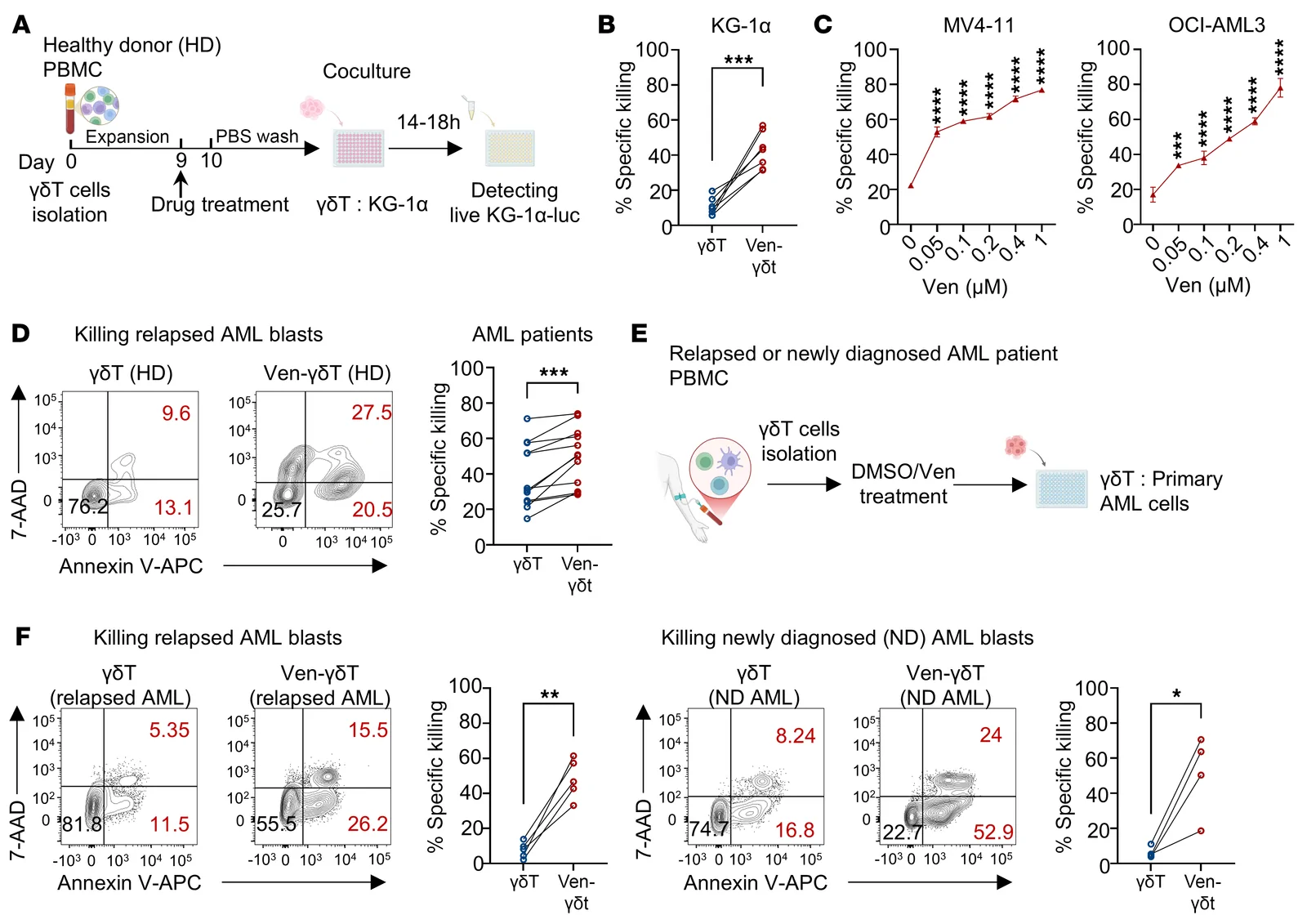
Venetoclax enhances the antileukemic activity of γδ T cells. (**A**) Schematic workflow for screening compounds that enhance γδ T cell function. (**B**) Killing efficiency of venetoclax-pretreated γδ T cells (ven-γδ T cells) against KG-1α cells. γδ T cells from 7 healthy donors were pretreated with dimethyl sulfoxide (DMSO) or 1 μM venetoclax for 18 hours and cocultured with KG-1α cells at a 1:1 effector-to-target (E:T) ratio for 14–18 hours. Cytotoxicity was determined by luminescence. Each paired symbol represents 1 donor (*n* = 7). (**C**) Dose-dependent killing efficiency of ven-γδ T cells against MV4-11 and OCI-AML3 cells at a 1:1 E:T ratio (*n* = 3 biological replicates). Data represent mean ± SD and were analyzed using repeated measures 1-way ANOVA with Dunnett’s multiple comparisons test. (**D**) Representative flow cytometry plots and quantification of ven-γδ T cell cytotoxicity against primary AML blasts from relapsed or newly diagnosed patients (*n* = 12 donors). Representative plots from 1 donor sample are shown. Cytotoxicity was assessed by annexin V/ 7-aminoactinomycin D (7-AAD) staining after 12 hours of coculture at a 1:1 E:T ratio. Data were analyzed using Wilcoxon’s matched pairs signed-rank test. (**E**) Schematic of cytotoxic assay using γδ T cells freshly derived from peripheral blood mononuclear cells (PBMCs) of relapsed or newly diagnosed patients with AML. (**F**) Cytotoxicity of patient-derived ven-γδ T cells against autologous AML blasts from relapsed AML patients (*n* = 5) and newly diagnosed AML patients (*n* = 4), assessed after 12 hours of coculture at a 4:1 E:T ratio. Data were analyzed using 2-tailed paired Student’s *t* test (**B** and **F**). *P < 0.05; **P < 0.01; ***P < 0.001; ****P < 0.0001.

Working with γδ T cells from healthy donors that we expanded with a standard protocol (32), we initially conducted a cytotoxicity assay to test killing activity against an AML cell line (KG-1α cells). We used an ex vivo venetoclax pretreatment (1 μM) of γδ T cells for 18 hours prior to the cytotoxicity assay (Figure 2A). Compared with the control (DMSO pretreatment) cells, our “ven-γδ T cells” showed a significantly increased extent of KG-1α cell killing (Figure 2B), a finding lending support to our speculation about potentially boosted therapeutic efficacy upon pretreatment of γδ T cells with venetoclax.

We conducted killing assays assessing 4 additional contexts: ex vivo pretreating of γδ T cells that we expanded from multiple healthy donors, using a diverse set of AML cell lines as target cells, using AML patient-derived primary AML blasts as target cells, and using primary γδ T cells from treatment-naive and relapsed AML patients targeting autologous AML blasts. Across these various assays, we consistently found that pretreating γδ T cells with venetoclax significantly boosted their cytotoxic capacity against AML target cells (Figure 2, C–F, and Supplemental Figure 1, A–C). A similar enhancement was also observed in Vδ1 γδ T cells, in which 1 μM venetoclax pretreatment increased cytotoxicity against KG-1α, MV4-11, and THP-1 cells (Supplemental Figure 2A). Importantly, this enhanced cytotoxicity was retained after cryopreservation and thawing of venetoclax-conditioned γδ T cells, supporting the feasibility of incorporating venetoclax conditioning into an off-the-shelf manufacturing workflow (Supplemental Figure 2B).

**Figure 3:**
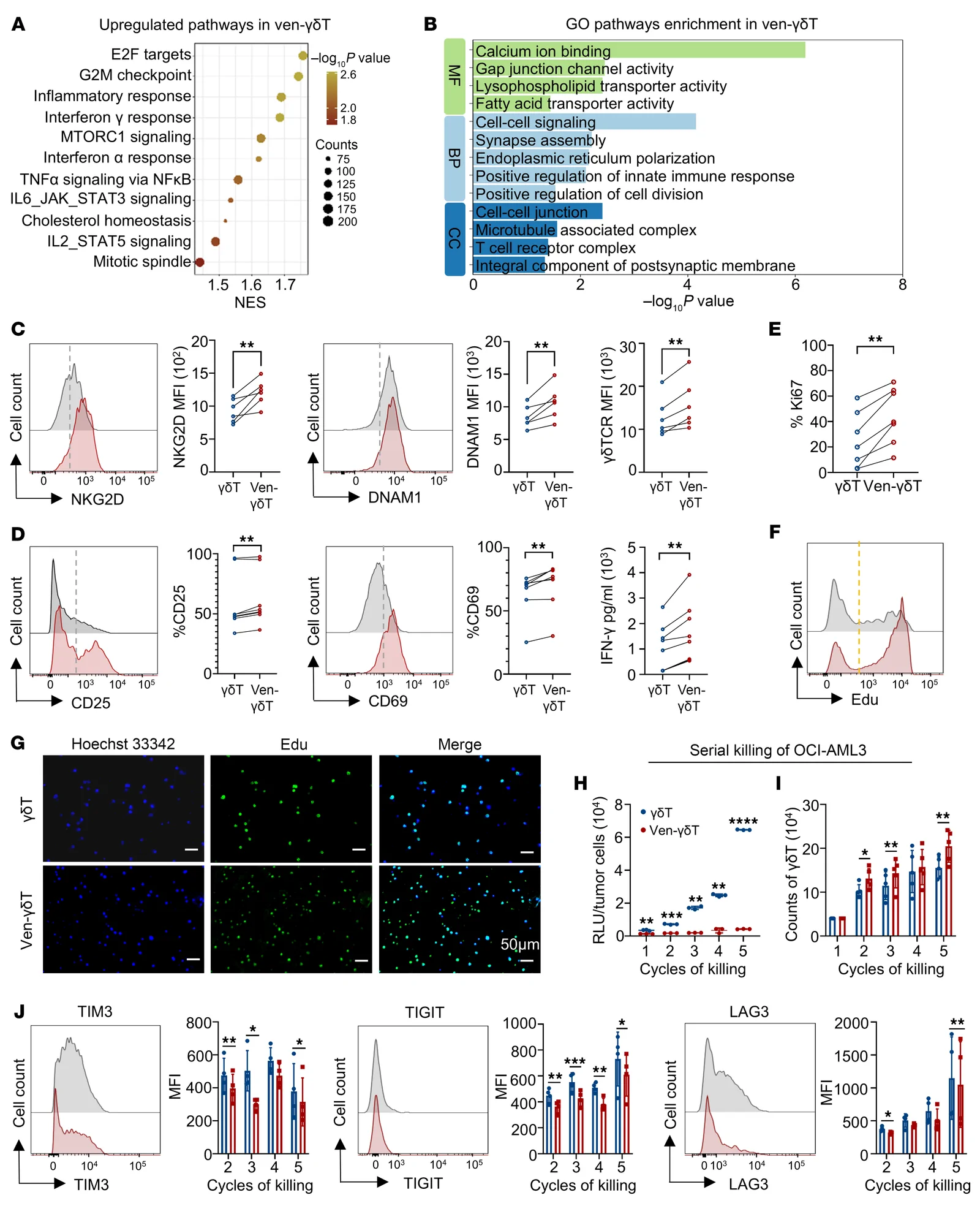
The venetoclax pretreatment promotes γδ T cell expansion and decreases exhaustion. (**A**) Representative upregulated gene set enrichment analysis pathway enrichment analysis in ven-γδ T cells. NES, normalized enrichment score. (**B**) Representative GO enrichment results for molecular function (MF), biological process (BP), and cellular component (CC) categories. (**C**) Median fluorescence intensity (MFI) of activating receptors, including natural killer group 2D (NKG2D), DNAX accessory molecule-1 (DNAM-1), and γδ TCR, on γδ T cells treated with dimethyl sulfoxide (DMSO) or venetoclax (1 μM, 18 hours), assessed by flow cytometry. Each paired symbol represents 1 donor (*n* = 6). (**D**) Expression of activation markers CD25 and CD69 on ven-γδ T cells after coculture with KG-1α cells, and IFN-γ levels in culture supernatants measured by ELISA. Each paired symbol represents 1 donor (*n* = 8). (**E**) Flow cytometric analysis of Ki-67 expression in γδ T cells after venetoclax treatment. Each point represents an independent donor (*n* = 7). (**F** and **G**) Flow cytometry plots of EdU incorporation (**F**) and representative immunofluorescence images of EdU and Hoechst 33342 staining (**G**). Images and plots are representative of 3 independent donor experiments. Scale bars: 50 μm. (**H**–**J**) Serial killing assays using γδ T cells expanded with DMSO or venetoclax (400 nM) and repeatedly challenged with OCI-AML3 cells. (**H**) AML viability by luminescence (*n* = 3 donors). (**I**) Absolute γδ T cell counts across killing cycles (*n* = 5 donors). (**J**) MFI of inhibitory receptors TIM3, LAG-3, and TIGIT during serial killing (*n* = 4 donors). Data represent the mean ± SD and were analyzed by 2-tailed paired Student’s *t* test. Data represent mean ± SD. Paired 2-tailed Student’s *t* test was used unless otherwise indicated. *P < 0.05; **P < 0.01; ***P < 0.001; ****P < 0.0001.

Notably, an RNA-seq analysis and a set of confirmatory follow-up analyses using flow cytometry showed trends consistent with increased cytotoxic capacity for the ven-γδ T cells, including significantly elevated levels of natural killer group 2D (NKG2D), DNAX accessory molecule-1 (DNAM-1), and γδ TCR (Figure 3, A–C). Moreover, the ven-γδ T cells showed stronger activation upon AML cell contact than the DMSO control group cells, as evidenced by elevated expression of CD69, CD25, and IFN-γ for analyte cells collected at the end of the assay (Figure 3D). These results demonstrate proof of concept that our ex vivo pretreatment of γδ T cells with venetoclax mirrors the increased anti-AML cytotoxic capacity known for a similar pretreatment of αβ T cells.

### The venetoclax pretreatment promotes γδ T cell proliferation and decreases exhaustion.

The aforementioned studies examining αβ T cells have repeatedly emphasized positive impacts of a venetoclax pretreatment on proliferation and expansion (9, 31), so it was notable that our RNA-seq analysis of ven-γδ T cells indicated elevated levels for genes with functional annotations related to cell division, the G2M checkpoint, and mitotic spindles, among others (Figure 3, A and B, and Supplemental Tables 4 and 5). We examined the proliferation of ven-γδ T cells with multiple assays: the ven-γδ T cells showed significantly increased proliferation over the DMSO control cells in both 5-ethynyl-2′-deoxyuridine (EdU)/propidium iodide (PI) and calcein AM assays (Figure 3, F and G, and Supplemental Figure 3, A and B), and flow cytometry showed that the ven-γδ T cells had significantly increased expression of the proliferation marker Ki-67 (Figure 3E). Additionally, brief exposure (18 hours) increased γδ T cell expansion, and extended culture with venetoclax further augmented proliferation without compromising viability (Supplemental Figure 3, C–F). Consistent with prior observations in NK cells that venetoclax sensitivity is influenced by activation state (33), activated/expanded γδ T cells showed reduced venetoclax-induced cell death compared with resting γδ T cells (Supplemental Figure 3G). We next assessed antiapoptotic protein expression and found higher myeloid cell leukemia 1 (MCL-1) and BCL-XL levels in activated γδ T cells than in resting cells. Under the same assay conditions, the MCL-1 inhibitor S63845 and the BCL-2/BCL-XL inhibitor navitoclax induced greater cell death than venetoclax at matched concentrations (Supplemental Figure 3, H and I). The activated/expanded γδ T cell population that survives the conditioning window thus displays the metabolic and functional features described below, irrespective of whether differential proliferation among γδ T cell subpopulations contributes to the recovered cell composition.

We subsequently conducted serial killing assays to assess the potential benefits of the venetoclax pretreatment in terms of killing persistence and exhaustion markers. Briefly, compared with control DMSO-γδ T cells, the ven-γδ T cells achieved superior antileukemic activity and higher proliferation across a panel of AML lines in up to 5 rounds of AML cell exposure (Figure 3, H and I, and Supplemental Figure 4, A–C), supporting that the pretreatment promotes the killing persistence of γδ T cells. Notably, the DMSO-γδ T cells showed significantly increased levels of exhaustion markers (e.g., T cell immunoglobulin mucin receptor 3 [TIM3], T cell immunoreceptor with Ig and ITIM domains [TIGIT], lymphocyte activation gene 3 [LAG-3]) compared with ven-γδ T cells, indicating that the venetoclax pretreatment somehow decreases the propensity of γδ T cells to undergo exhaustion after repeatedly engaging target cells for killing (Figure 3J). Further, we found that the venetoclax pretreatment led to a marked increase in the number of γδ T cells with a stem cell–like memory T cell phenotype (CD45RA^+^CD62L^+^, CCR7^+^; Supplemental Figure 4D). These findings establish that the venetoclax pretreatment promotes γδ T cell proliferation and reduces functional exhaustion after the ven-γδ T cells repeatedly engage target AML cells for killing.

### The venetoclax pretreatment boosts the in vivo efficacy of a γδ T cell therapy against AML.

**Figure 4:**
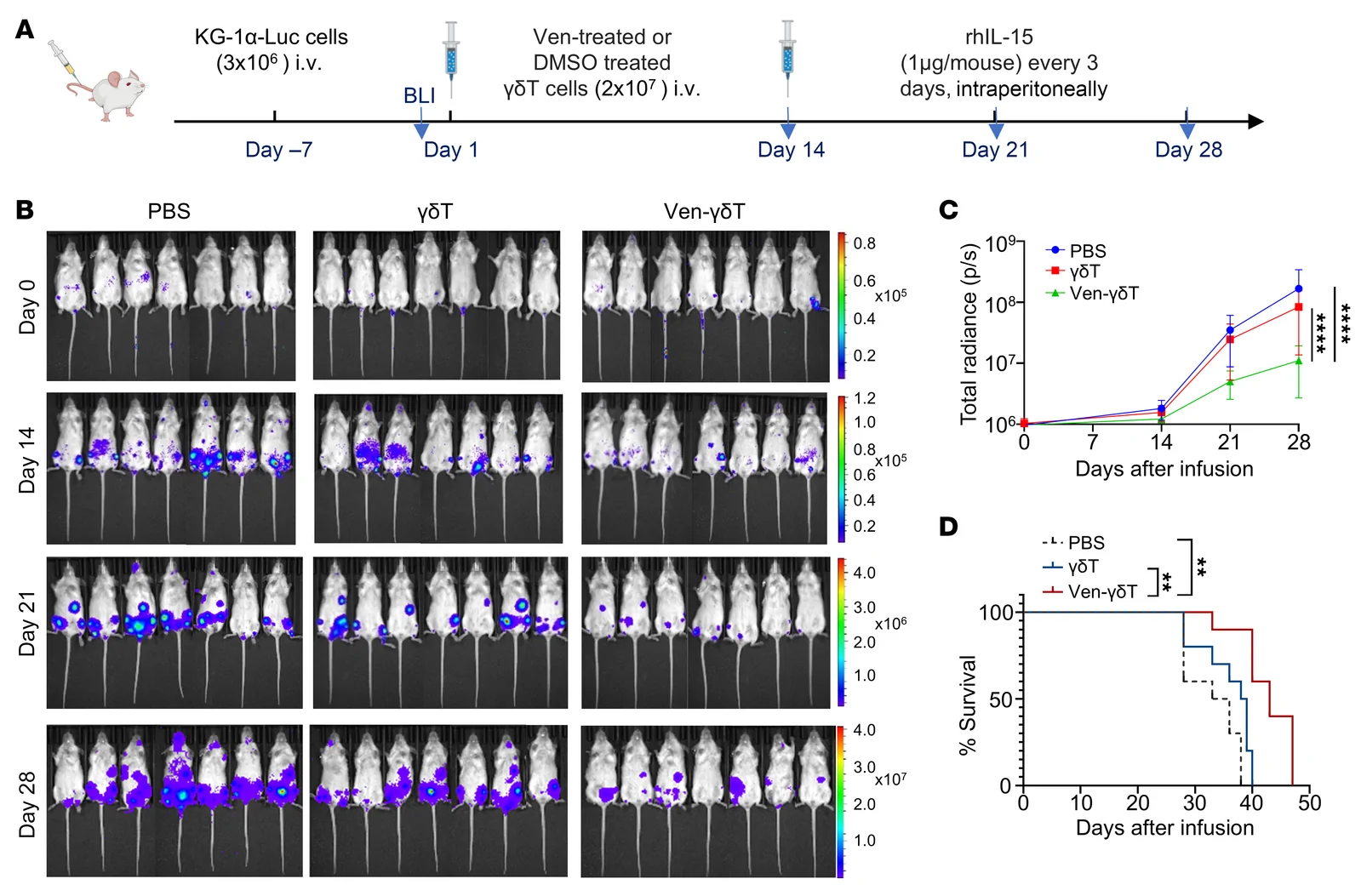
Venetoclax enhances the in vivo efficacy of γδ T cells against AML. (**A**) Schematic of the experimental procedure. NPG mice were engrafted with 3 × 10^6^ KG-1α-luc cells. One week later, mice were treated with vehicle control, DMSO-γδ T cells, or ven-γδ T cells (2 × 10^7^ cells per infusion, 2 infusions, 2-week interval). KG-1α is an immature AML cell line with complex cytogenetic abnormalities, including del(7) and multiple monosomies, and a reported TP53 alteration. (**B**) Bioluminescence images of tumor burden in 3 groups. (**C**) Quantification of bioluminescence imaging values representing tumor burden, *n* = 7. Data represent mean ± SD. Statistical significance was determined using 2-way repeated measures ANOVA with Tukey’s multiple comparisons test. ****P < 0.0001. (**D**) Kaplan-Meier survival analysis (*n* = 7 mice per group). Statistical significance was determined by the log-rank test. **P < 0.01.

In light of the clinical data supporting differential γδ T cell abundance and functional phenotypes in the CR versus relapsed AML patients, and encouraged by our discovery that a venetoclax pretreatment boosts γδ T cell activity against AML blasts, we next examined potential in vivo therapeutic benefits from ven-γδ T cells against AML. We established a xenograft model using NOD/Prkdcscid/IL2rgnull (NPG) immunodeficient mice: mice were engrafted with KG-1α-luc cells and were subsequently given adoptive transfer of ven-γδ T cells (2 × 10^7^ cells/mouse, twice at a 2-week interval; recombinant human IL-15 [rhIL-15] 1 μg/mouse, every 3 days), γδ T cells with DMSO pretreatment, or PBS (Figure 4A).

**Figure 5:**
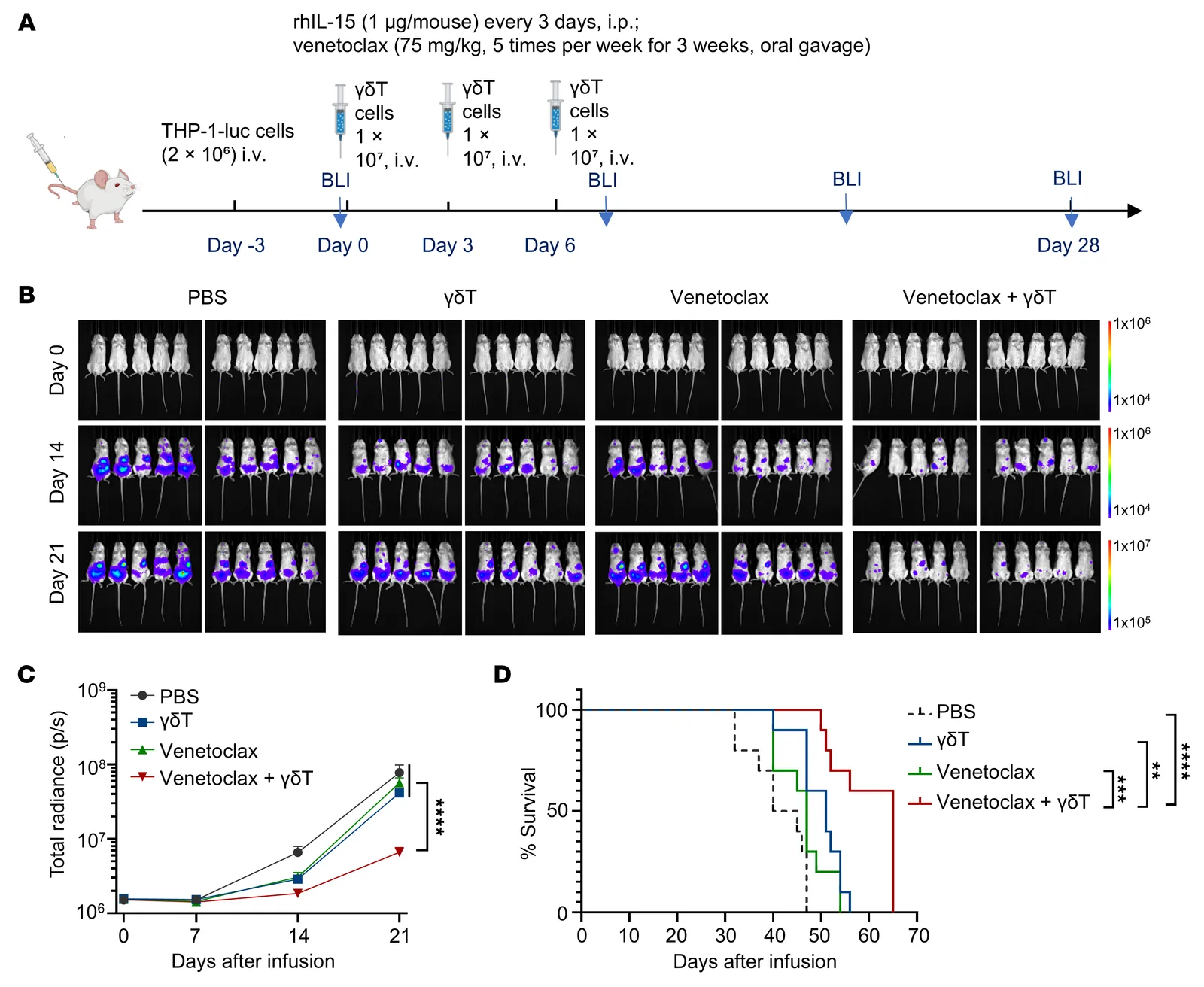
Venetoclax gavage augments the in vivo efficacy of adoptively transferred γδ T cells against AML. (**A**) Schematic of the experimental procedure. NPG mice were inoculated intravenously with THP-1-luciferase AML cells. Three days after tumor engraftment, mice were randomized into 4 groups: (i) PBS control; (ii) venetoclax alone (75 mg/kg by oral gavage, 5 times per week for 3 weeks); (iii) γδ T cell infusion alone (1 × 10^7^ cells per mouse via tail vein, once every 3 days, total of 3 infusions); and (iv) combination venetoclax + γδ T cells (same venetoclax schedule plus γδ T cell infusions as above). Tumor burden was monitored longitudinally by bioluminescence imaging. THP-1 is a human acute monocytic leukemia (FAB M5) cell line with reported oncogenic NRAS gain-of-function and TP53 alterations. (**B**) Bioluminescence imaging of mice (*n* = 10). (**C**) Quantification of bioluminescence imaging values representing tumor burden (*n* = 10 mice per group). Data represent mean ± SD. Statistical significance was determined using 2-way repeated measures ANOVA with Tukey’s multiple comparisons test. ****P < 0.0001. (**D**) Kaplan-Meier survival analysis of THP-1-luc–engrafted NPG mice treated with PBS, γδ T cells, oral venetoclax, or oral venetoclax plus γδ T cells (*n* = 10 mice per group). Statistical significance was determined by the log-rank test. **P < 0.01; ***P < 0.001; ****P < 0.0001.

Supporting an antileukemia benefit from the venetoclax pretreatment, the extent of KG-1α engraftment was significantly reduced in the ven-γδ T cell group compared with both the γδ T cell and PBS groups (Figure 4, B and C). Consistent with this enhanced leukemia control, mice treated with ven-γδ T cells showed a significantly prolonged survival compared with those receiving DMSO-pretreated γδ T cells or PBS (Figure 4D). To model a clinically applicable regimen, we further tested oral venetoclax in THP-1-luc–engrafted NPG mice randomized to receive PBS, γδ T cells, oral venetoclax, or the combination (Figure 5A). Oral venetoclax plus γδ T cells achieved superior leukemia control over either monotherapy (Figure 5, B and C). Notably, the combination group also exhibited significantly prolonged survival relative to all other treatment groups, supporting an in vivo benefit of combining venetoclax with γδ T cell therapy in this model (Figure 5D).

Collectively, the results from experiments targeting AML blasts and AML model animals demonstrate that our strategy of pretreating γδ T cells with venetoclax clearly increases their antileukemic activity, to the extent of conferring a therapeutic benefit.

### Venetoclax pretreatment enhances mitochondrial abundance and promotes FAO–OXPHOS–linked metabolism in γδ T cells.

**Figure 6:**
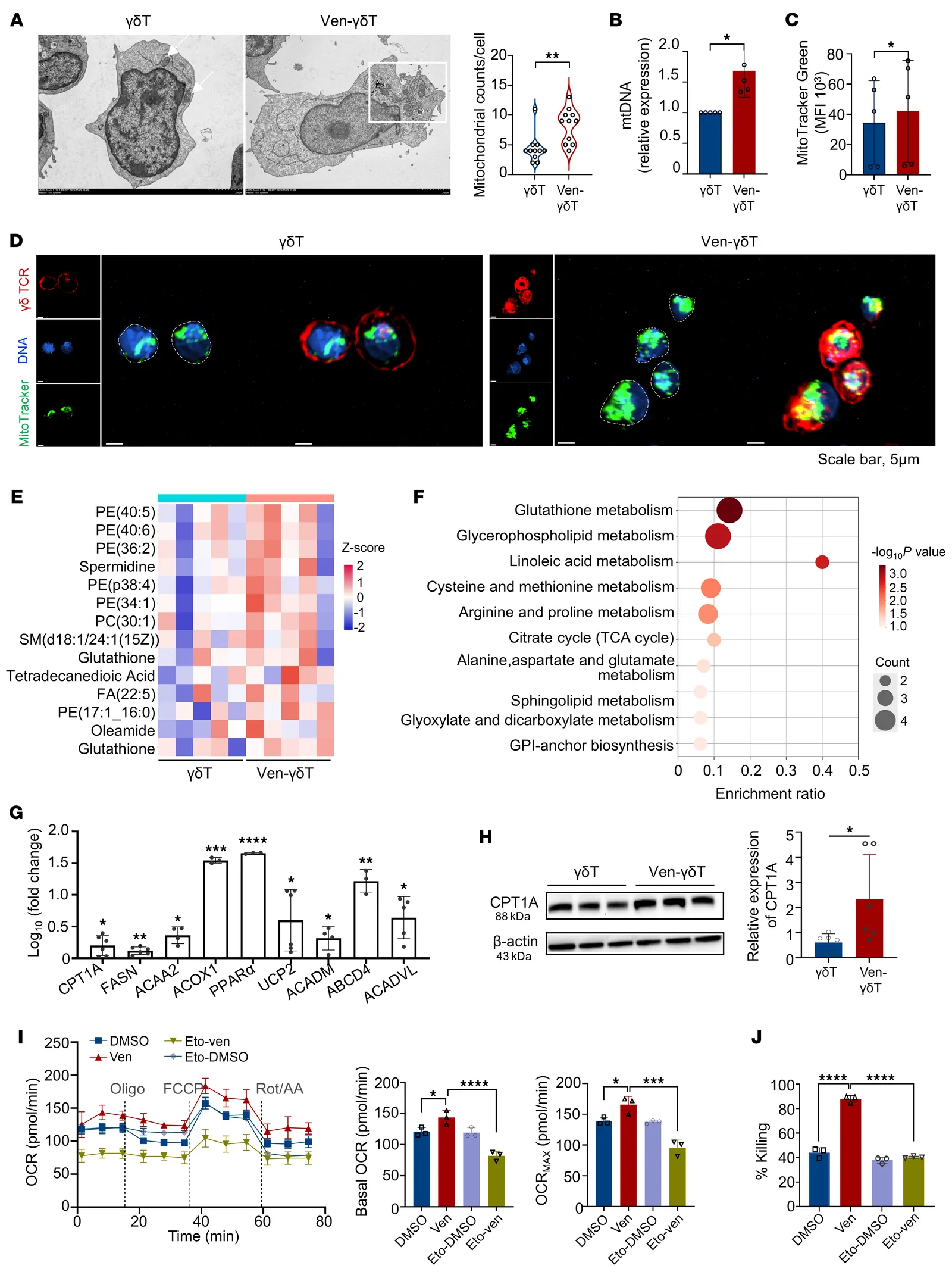
Venetoclax promotes mitochondrial abundance and FAO–OXPHOS–linked metabolism in γδ T cells. (**A**) Transmission electron microscopy of γδ T cells pretreated with dimethyl sulfoxide (DMSO) or venetoclax. White arrows indicate mitochondria. Scale bars: 2 μm. Mitochondrial number was quantified from more than 3 randomly selected cells per group (*n* = 3 donors). (**B**) Mitochondrial DNA content in DMSO- and venetoclax-pretreated γδ T cells (ven-γδ T cells), measured by qPCR for mitochondrially encoded cytochrome c oxidase I (MT-CO1), as a marker of mitochondrial DNA content, relative to β-actin (*n* = 5 donors). (**C**) Mitochondrial mass assessed by MitoTracker Green flow cytometry (*n* = 5 donors). (**D**) Confocal images and 3D renderings of DMSO- and ven-γδ T cells. Images are representative of 3 independent donor experiments. Red, γδ TCR; blue, Draq5; green, MitoTracker Green. Scale bars: 5 μm. (**E**) Heatmap of differentially abundant metabolites (*P* < 0.05) identified by untargeted metabolomics using γδ T cells from 5 donors. Abundances are *Z*-score–normalized. (**F**) Pathway enrichment by MetaboAnalyst; main pathways with a *P* value < 0.05 are shown. (**G**) Fold-changes of FAO-related genes in ven-γδ T cells versus DMSO-γδ T cells, normalized to β-actin (≥3 donors, triplicate measurements). (**H**) CPT1A protein by immunoblot (representative of 3 donors) with pooled quantification (*n* = 6 donors). Mean ± SD. Data were analyzed using Wilcoxon’s matched pairs test. (**I**) OCR curves after DMSO, venetoclax, etomoxir (Eto) plus DMSO, or venetoclax plus etomoxir (5 μM, 18 hours) treatment, with basal and maximal OCR quantified. Data are representative of 3 independent donor experiments. Oligo, oligomycin; Rot/AA, rotenone and antimycin A; FCCP, carbonyl cyanide *p*-trifluoromethoxyphenylhydrazone. (**J**) Cytotoxicity of γδ T cells after the indicated treatments (*n* = 3). Data represent mean ± SD. Statistical analyses were performed using unpaired, 2-tailed Student’s *t* test (**A** and **G**), paired 2-tailed Student’s *t* test (**B** and **C**), and repeated measures 1-way ANOVA with Tukey’s multiple comparisons test (**I** and **J**). *P < 0.05; **P < 0.01; ***P < 0.001; ****P < 0.0001.

We next conducted a series of analyses to investigate how the venetoclax pretreatment promotes γδ T cell proliferation and reduces functional exhaustion. We first conducted transmission electron microscopy analysis and observed a significantly higher mitochondria number in ven-γδ T cells, without affecting the morphology of mitochondria (Figure 6A and Supplemental Figure 5, A and B). Further, mitochondrial DNA (mtDNA) quantification showed significantly higher mitochondrial content in the ven-γδ T cells over control cells (Figure 6B), and assessment of mitochondrial mass (by MitoTracker Green) showed a significant increase in ven-γδ T cells (Figure 6, C and D). These findings together support that the venetoclax pretreatment somehow promotes mitochondrial abundance.

We also conducted untargeted metabolomic profiling that revealed substantially increased levels of multiple lipid species (e.g., unsaturated phospholipids, long-chain unsaturated fatty acids) in ven-γδ T cells over DMSO-γδ T cells (Figure 6E and Supplemental Table 6). Additionally, pathway enrichment analysis identified FAO-related metabolic pathways in ven-γδ T cells (Figure 6F). FAO-related transcripts were upregulated (Figure 6G), and carnitine palmitoyltransferase 1A (CPT1A), the rate-limiting enzyme of FAO, was also elevated at the protein level (Figure 6H). Considering these mitochondria and energy phenotypes together, we next monitored mitochondrial respiration in ven-γδ T cells and DMSO-γδ T cells after coculturing with AML blasts. The values for both the oxygen consumption rate (OCR) and spare respiratory capacity were significantly higher in ven-γδ T cells (Supplemental Figure 5C). Notably, despite this increased overall mitochondrial respiration, we detected no change in glycolytic activity as assessed by extracellular acidification rate (Supplemental Figure 5D).

Pursuing the differential OXPHOS profiles alongside the FAO-related changes, we examined ven-γδ T cells and DMSO-γδ T cells after exposure to an inhibitor of the FAO pathway enzyme CPT1A (etomoxir). FAO pathway inhibition attenuated the aforementioned venetoclax pretreatment–induced increases in OCR (Figure 6I) and led to an approximately 40% reduction in the magnitude of the venetoclax pretreatment–induced boost in the cytotoxic capacity of γδ T cells (Figure 6J). Collectively, these results support that the venetoclax pretreatment promotes an FAO–OXPHOS–skewed form of energy metabolism in γδ T cells and moreover that this FAO–OXPHOS–skewed metabolic impact of the venetoclax pretreatment can at least partially account for the increased proliferation and decreased exhaustion observed in ven-γδ T cells.

### Venetoclax enhances CLL-1 CAR γδ T cell–mediated cytotoxicity against AML in vivo.

**Figure 7:**
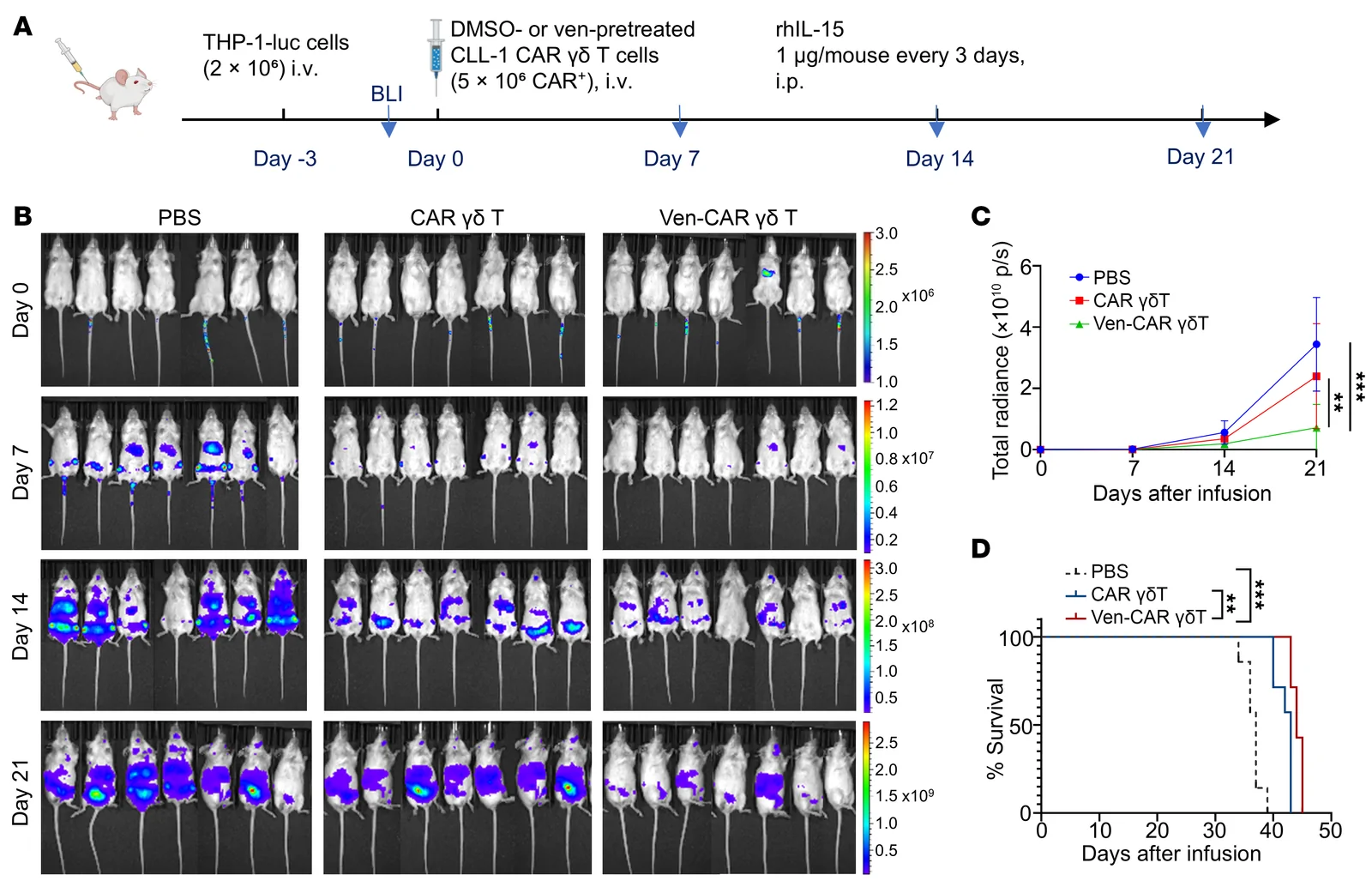
Venetoclax enhances the in vivo efficacy of CLL-1 CAR γδ T cells against AML. (**A**) Schematic of the experimental procedure. NPG mice were intravenously engrafted with 2 × 10^6^ THP-1-luc cells. Three days later, mice were treated with vehicle control, DMSO-CAR γδ T cells, ven-CAR γδ T cells (5 × 10^6^ CAR^+^ cells per mouse). (**B**) Bioluminescence images of tumor burden in 3 groups. (**C**) Quantification of tumor burden, *n* = 7 mice per group. Data represent mean ± SD. Statistical significance was determined using 2-way repeated measures ANOVA with Tukey’s multiple comparisons test. **P < 0.01; ***P < 0.001. (**D**) Kaplan-Meier survival analysis (*n* = 7 mice per group). Statistical significance was determined by the log-rank test. **P < 0.01; ***P < 0.001.

Given the in vivo therapeutic benefit we observed for ven-γδ T cells, and considering the numerous advantages that are possible with engineered cell therapies like CAR T cells, we next explored whether a venetoclax pretreatment can increase the efficacy of therapeutic CAR γδ T cells. Specifically, we engineered CAR γδ T cells targeting CLL-1, a surface antigen that is highly expressed on THP-1 cells (34). Mice engrafted with THP-1 cells were given PBS or treated with adoptive transfer of these CAR γδ T cells or venetoclax-pretreated CAR γδ T cells (Figure 7A).

Consistent with our findings from the earlier in vivo efficacy experiments using adoptive transfer of γδ T cells (i.e., nonengineered), our ven-CAR γδ T cells led to a significantly reduced extent of AML engraftment compared with both the PBS and CAR γδ T cell groups (Figure 7, B and C). Ven-CAR γδ T cells also showed a significantly prolonged survival compared with those receiving DMSO-pretreated CAR γδ T cells or PBS (Figure 7D). These results collectively establish that the ex vivo venetoclax pretreatment boosts the efficacy of both therapeutic γδ T cells and therapeutic CAR γδ T cells.

## Discussion

The present study shows that short-term pharmacological inhibition of BCL-2 enhances both the metabolic and functional properties of therapeutic γδ T cells. Venetoclax pretreatment increased cytotoxic activity, proliferation, and persistence while reducing exhaustion and was associated with elevated mitochondrial content and an FAO–OXPHOS–linked metabolic profile. In vivo testing demonstrated that venetoclax-pretreated γδ T cells achieved stronger antileukemic responses in AML models, indicating improved therapeutic efficacy. However, these xenograft results should be interpreted cautiously, as the nominal E:T ratios used in immunodeficient mouse models are not directly clinically feasible and therefore provide proof of concept rather than a direct representation of clinical dosing conditions. Extending this approach to CLL-1–directed CAR γδ T cells also enhanced antileukemic efficacy, supporting that venetoclax as a pretreatment can boost the efficacy of γδ T cell–based therapies and potentially inform the design of optimized immunotherapeutic strategies for AML. In a related clinical context, the EVICTION study reported high remission rates in newly diagnosed AML patients receiving the anti-BTN3A antibody ICT01 combined with azacitidine and venetoclax (35). The EVICTION regimen activates endogenous Vγ9Vδ2 T cells in vivo during concurrent venetoclax exposure, whereas the present study tests short-term ex vivo venetoclax conditioning of expanded γδ T cells before adoptive transfer. Despite these mechanistic differences, both observations support the inference that γδ T cell antileukemic function can be productively engaged during or after venetoclax exposure rather than abrogated by it, consistent with the activation state–dependent venetoclax sensitivity observed in the present study (Supplemental Figure 3G).

Several studies have linked oxidative metabolism to the persistence and proliferative capacity of therapeutic T cells. In αβ T cells, interventions that increase OXPHOS — such as IL-10 exposure (36), IDH2 inhibition (13), or mitogen-activated protein kinase kinase 1/2 blockade (37) — promote sustained effector function and delay exhaustion. Related work showed that the monounsaturated fatty acid oleic acid restores γδ T cell cytotoxicity under lipid-induced stress (38). In our study, venetoclax pretreatment produced γδ T cells with elevated mitochondrial mass and an FAO–OXPHOS metabolic profile, consistent with these established pathways. These findings indicate that BCL-2 inhibition acts within a broader class of metabolic interventions that support long-term function through oxidative metabolism. Future work should determine whether the benefits of this conditioning reflect a direct metabolic shift or an indirect coupling between apoptotic priming and mitochondrial remodeling. We note that etomoxir at concentrations above ~3–10 μM has been reported to engage off-target effects on the adenine nucleotide translocase and on broader mitochondrial function in addition to CPT1A inhibition; our interpretation of the etomoxir results in Figure 6, I and J, is accordingly scoped to functional dependence on the CPT1A-containing FAO pathway under the tested assay conditions rather than to exclusive CPT1A target engagement.

Enhanced FAO in the venetoclax-pretreated γδ T cells also resembles the energy utilization pattern of stem-like and central memory T cells, which rely on FAO to sustain self-renewal and persistence (39). This convergence suggests that pharmacological modulation of apoptosis may promote a memory-like metabolic program in γδ T cells without genetic modification. Determining whether this metabolic state corresponds to a discrete differentiation phenotype or a reversible functional adaptation would be informative. Such studies could define whether short-term metabolic conditioning can be combined with existing cytokine or signaling-based manufacturing protocols to optimize γδ T cell persistence and antitumor efficacy across multiple therapeutic settings.

Looking forward, these findings support a broader research initiative to explore whether γδ T cell products — now advancing toward clinical translation — can benefit from the expanding toolkit of metabolic and apoptotic modulators that have already enhanced αβ T cell therapies. In αβ T cells, small molecule inhibitors targeting pathways such as PI3K, AKT, and IDH2 have been shown to enhance persistence, reduce exhaustion, and improve functional phenotypes during manufacturing. However, the extent to which these same interventions might tune the distinct metabolic wiring and activation states of γδ T cells remains unknown. Given the divergent lineage programs and innate-like activation of γδ T cells, it is unlikely that these agents will exert identical effects. In αβ T cells, venetoclax exposure has mostly been reported to reshape subset composition (7, 40) (depleting BCL-2–dependent naive cells, enriching effector-memory pools) and increase activation receptors or ROS (8), but often without a parallel increase in OXPHOS, and in some settings with restoration of glycolysis driven by tumor debulking rather than direct mitochondrial remodeling (41). Still, they may prove valuable in optimizing γδ T cell function or persistence in therapeutic contexts. This work opens the door for systematic efforts to define how metabolic reprogramming strategies can be adapted — or newly developed — for γδ T cell–based immunotherapies.

## Methods

### Sex as a biological variable.

Both sexes were included in clinical sample analyses and animal studies to ensure comprehensive representation. The findings are anticipated to be applicable to both sexes.

### scRNA-seq reanalysis.

Public scRNA-seq data from Mathioudaki et al. were reanalyzed (19). Primary post–allo-HSCT BM samples were Ficoll-separated, sorted into CD34^+^ and CD3^+^ fractions, and profiled with 10x Genomics Chromium Single Cell 3′ Gene Expression v3 assays on an Illumina NextSeq 500 instrument. Processed matrices (Cell Ranger) were downloaded from the authors’ repository (https://github.com/ZauggGroup/scBM_alloSCT; commit ID f74c153). Our analyses focused on γδ T cells identified by canonical transcripts (e.g., TRGC1, TRDC) and standard quality control thresholds. Differential expression between patient subgroups was performed in Seurat v4 (R) using thresholds of |log fold-change| > 0.25, *P* < 0.05, and min.pct > 0.1. KEGG and GO enrichment analyses were conducted for the differentially expressed genes.

### γδ T cell isolation and venetoclax conditioning.

For γδ T cell isolation from patient PBMCs, CD3^+^ T cells were enriched by negative selection, then positively selected with anti-TCRγδ microbeads (130-050-701, Miltenyi Biotec). Frozen γδ T cells and CLL-1 CAR γδ T cells expanded from healthy donors (UnicetBio) were thawed at 37°C (1–2 minutes), diluted 1:10 in RPMI-1640 with 10% FBS, centrifuged (400*g*, 10 minutes), resuspended at 1 × 10^6^ cells/mL with human IL-2 (100 IU/mL), and rested 24 hours. Purity (>80%) was confirmed by flow cytometry (anti–TCR Vδ2, anti-CD3) (see Supplemental Methods “Flow cytometry”).

For short-term conditioning, donor γδ T cells were treated with venetoclax (1 μM; ABT-199, MedChemExpress) for 18 hours; AML patient-derived γδ T cells were treated with 400 nM venetoclax for 18 hours. Conditioning concentrations were selected based on cell death assessments across venetoclax concentrations for each cell source to minimize treatment-associated cell death (Supplemental Figure 1, D and E, and Supplemental Figure 3G). Before cytotoxicity assays, cells were washed twice with PBS and resuspended in assay medium.

γδ T cells were expanded as previously described (32). Briefly, PBMCs from leukapheresis (Ficoll) were cultured in good manufacturing practice–compliant (GMP–compliant) serum-free medium supplemented with rhIL-2 (200 U/mL) and l-glutamine (2 mM) (Gibco). Zoledronic acid (5 μM, Sigma-Aldrich) was added on day 0 at 2 × 10^6^ cells/mL. Cultures were maintained at 37°C, 5% CO_2_, and adjusted to 1 × 10^6^ cells/mL every 2–3 days. Venetoclax (400 nM) was added at each medium change (venetoclax-conditioned expansion). On day 9, cell number and purity were assessed by flow cytometry.

### In vitro short-term and serial killing assays.

For short-term cytotoxicity against AML cell lines, DMSO- or venetoclax-pretreated γδ T cells (DMSO-γδ T or ven-γδ T) were cocultured with luciferase-expressing KG-1α, THP-1, OCI-AML3, or MV4-11 targets (2 × 10^4^ cells/well) (see Supplemental Methods “Human AML cell lines”) in RPMI-1640 with 10% FBS at the etomoxir E:T ratios detailed in Figure 2. After 14–18 hours, luminescence was measured using Bright-Lite luciferase substrate (Vazyme) on a SpectraMax iD5 luminometer. Percentage cytotoxicity was calculated relative to tumor-only controls as (RLU_tumor-only − RLU_co-culture) / RLU_tumor-only × 100%.

For primary AML blasts, DMSO-γδ T or ven-γδ T cells were labeled with CellTrace Violet (Thermo Fisher Scientific), then cocultured for 12 hours at E:T = 1:1 for healthy donor-derived assay or 4:1 for autologous patient-derived assay. Cells were then stained with anti-CD45 (see Supplemental Methods “Flow cytometry”), annexin V, and 7-AAD (BioLegend), then analyzed by flow cytometry. Specific killing was computed as %annexin V^+^ (with γδ T) − %annexin V^+^ (without γδ T), normalized to [100 − %annexin V^+^ (without γδ T)].

For serial killing assays, γδ T cells expanded with venetoclax (400 nM) were cocultured with luciferase-labeled AML targets at an initial E:T = 4:1; equal numbers of fresh targets were added every 24 hours. Tumor luminescence was recorded every 24 hours. Total cells were counted (Countstar Mira FL), and absolute γδ T cell counts were calculated as γδ T cell percentage by flow cytometry × total cell number.

### In vivo xenograft studies and bioluminescence imaging.

NPG mice (6–8 weeks; including both male and female mice, Vitalstar) were used for all xenograft studies. For the KG-1α-luc model, mice were intravenously injected with 3 × 10^6^ KG-1α-luc cells. On day 7 after tumor inoculation, mice received 2 × 10^7^ DMSO-pretreated or venetoclax-pretreated γδ T cells by tail vein injection, followed by a second infusion 14 days later. For the THP-1-luc γδ T cell model, mice were intravenously injected with 2 × 10^6^ THP-1-luc cells. On day 3 after tumor inoculation, mice were assigned to 4 groups: PBS control, γδ T cells alone, venetoclax alone, or venetoclax combined with γδ T cells. Mice in the γδ T cell treatment groups received 1 × 10^7^ γδ T cells by tail vein injection every 3 days for a total of 3 infusions. Venetoclax was administered by oral gavage at 75 mg/kg, 5 times per week for 3 weeks, starting on the first day of γδ T cell infusion. For the THP-1-luc CLL-1 CAR γδ T cell model, mice were intravenously injected with 2 × 10^6^ THP-1-luc cells. On day 3 after tumor inoculation, mice received a single tail vein infusion of 5 × 10^6^ CAR^+^ CLL-1 CAR γδ T cells.

In all models, rhIL-15 (1 μg/mouse) was administered intraperitoneally starting on the day of γδ T cell infusion and then every 3 days thereafter. Body weight and clinical status (ruffled fur, decreased activity, hunched posture, labored breathing) were monitored; mice were euthanized at predefined humane endpoints (>15% weight loss or clinical decline). For assessment of tumor burdens, mice received d-luciferin (15 mg/kg, intraperitoneal) 10 minutes before imaging on an IVIS Lumina III (PerkinElmer). Images (10 minute exposure) were quantified as radiance (photons/s/cm^2^/sr) in Living Image v4.7.3.

### Statistics.

For comparisons between 2 groups, 2-tailed Student’s *t* tests were used for parametric unpaired data, with Welch’s correction applied when variances were unequal; paired 2-tailed Student’s *t* tests or Wilcoxon matched pairs signed-rank tests were used for paired data as appropriate. For comparisons involving more than 2 groups, repeated measures 1-way ANOVA followed by Tukey’s or Dunnett’s multiple comparisons test was used as indicated in the figure legends. Longitudinal bioluminescence imaging data were analyzed using 2-way repeated measures ANOVA followed by Tukey’s multiple comparisons test. Survival was analyzed using the log-rank Mantel-Cox test. No samples were excluded from analysis unless otherwise stated. Data are presented as mean ± SD unless otherwise indicated. A *P* value less than 0.05 was considered statistically significant.

### Study approval.

Primary BM or peripheral blood samples from patients with AML were obtained at the Department of Hematology, First Affiliated Hospital of USTC (Hefei, China), after receipt of written informed consent, in accordance with the Declaration of Helsinki and with approval from the Ethics Committee of the First Affiliated Hospital of USTC (2024KY-041). Sample characteristics are summarized in Supplemental Table 7. All animal procedures were approved by the Ethics Committee of the First Affiliated Hospital of USTC [2023-N(A)-0189].

### Data availability.

RNA-seq data generated in this study are publicly available in the National Center for Biotechnology Information Gene Expression Omnibus under accession number GSE306856. Values for all data points shown in graphs are provided in the Supporting Data Values file. Additional methods are provided in the Supplemental Methods.

## Author contributions

XC designed the study, performed experiments, collected whole-blood samples and clinical data, analyzed data, and wrote the manuscript. XC, LZ, BY, YW, and KY performed experiments and interpreted data. YS, WM, HZ, and JM assisted with data analysis and supplied cell products and γδ T cell culture reagents. YZ and BT helped conceive the study and provided mentorship. XZ designed the study, supervised the research, and revised the manuscript. All authors reviewed and approved the final manuscript.

## Conflict of interest

YS, WM, and HZ are employees of UnicetBio Co., Ltd., where WM serves as chief technology officer and HZ is the founder and chief executive officer. HZ and YZ hold equity interests in UnicetBio Co., Ltd.; YZ is also a founder of the company.

## Funding support

- National Natural Science Foundation of China (U23A20453 and 82270223 to XZ, 82170209 to BT, 82350109 and 32530039 to YZ).
- Anhui Provincial Department of Education Scientific Research Project (2023AH010079 to XZ).
- Anhui Provincial Natural Science Foundation (2308085J09 to XZ).
- USTC Research Funds of the Double First-Class Initiative (YD9110002047 to XZ).
- International Cooperation Projects in Anhui Province (2023h11020005 to BT).
- Research Funds of Centre for Leading Medicine and Advanced Technologies of IHM (2025IHM01080 to XZ).

## Supplementary Material

Supplemental data

Unedited blot and gel images

Supplemental tables 1-8

Supporting data values

## Version 1 07/23/2026

In-Press Preview

## Version 2 09/15/2026

Electronic publication
