# Supplementary material for: Ex vivo pretreatment with venetoclax boosts antileukemic efficacy of therapeutic γδ T cells in AML models
Source: J Clin Invest. 2026 Jul 23;136(18):e202932. doi: 10.1172/JCI202932 (PMC13574141; doi:10.1172/JCI202932)
Supplement: Supplementary file 1 [file jci-136-202932-s153.pdf]

Data Supplement

Manuscript Title: Ex vivo pretreatment with venetoclax boosts antileukemic efficacy of therapeutic γδ T cells in AML models

Authors: Xingchi Chen, Lin Zhang, Bingbing Yan, Yinqiang Sui, Weiwei Ma, Hui Zhao, Yining Wang, Kepeng Yang, Jiewen Ma, Baolin Tang, Yonghui Zhang, Xiaoyu Zhu

Supplemental methods:

Human AML cell lines

Human AML luciferase-expressing cell lines, including KG-1α-luc, THP-1-luc, OCI-AML3-luc, and MV4-11-luc cells, were kindly provided by UnicetBio Co., Ltd. KG-1α-luc and THP-1-luc cells were maintained in RPMI 1640 medium (Biosharp, Cat# BL303A), with 10% FBS (Sigma, Cat# F7524) and 1% penicillin-streptomycin (Thermo Fisher Scientific, Cat# 15140122). OCI-AML3-luc cells were maintained in RPMI 1640 supplemented with 20% FBS, 1 mM sodium pyruvate (Thermo Fisher Scientific, Cat# 11360070), 2 mM L-glutamine (Thermo Fisher Scientific, Cat# 25030081), 10 mM HEPES (Thermo Fisher Scientific, Cat# 15630130) and 1% penicillin-streptomycin. MV4-11-luc cells were cultured in IMDM (Thermo Fisher Scientific, Cat# 12440053) with 10% FBS and 1% penicillin-streptomycin. All cell lines were maintained at 37°C, supplemented with 5% CO_2.

Colony forming assays

To assess clonogenic growth after co-culture with γδ T cells or venetoclax-treated γδ T cells (ven-γδ T), KG-1α, OCI-AML3, MV4-11, or primary AML cells were pretreated for 12–14 h at an effector-to-target (E:T) ratio of 1:1. Subsequently, 2×10^4 cells per well were plated in 6-well plates in 1 mL MethoCult H4434 medium (StemCell Technologies, Cat# H4434). The plates were incubated at 37°C with 5% CO_2. After 12–14 days, colonies were counted under an inverted microscope. Clusters containing ≥10 cells were defined as a colony.

Generation of CLL-1 CAR γδ T cells

CLL-1 CAR γδ T cells were generated from healthy donor-derived Vγ9Vδ2 T cells using a lentiviral transduction protocol established in our laboratory and similar to that previously used for the generation of B7-H3 CAR-modified γδ T cells (1). Briefly, CAR construct was cloned into an HIV-1-derived lentiviral vector. The CAR consisted of a mouse Ig kappa leader sequence, a CLL-1-specific single-chain variable fragment (scFv), a CD8α hinge and transmembrane region, the intracellular costimulatory domain of 4-1BB, and the CD3ζ signaling domain. The CLL-1-specific scFv was derived from the anti-CLL-1 monoclonal antibody clone 1075.7, as previously reported (2), with the heavy- and light-chain variable regions linked by a (GGGGS)3 linker. A truncated epidermal growth factor receptor (EGFRt) was co-expressed downstream of the CAR sequence via a P2A peptide to enable monitoring of CAR expression. Expanded γδ T cells were transduced with the CLL-1 CAR lentiviral vector on day 1 of culture, and CAR expression was confirmed by flow cytometry prior to cryopreservation and functional assays.

Flow cytometry

For phenotyping and counting, samples were acquired on a BD LSR II instrument and analyzed using FlowJo v10.x (BD). CountBright absolute counting beads (Thermo Fisher) were used for absolute quantification where indicated. The following anti-human antibodies were used for cell staining: CD3 (SK7)-APC Cy7 (Cat# 560176) or -FITC (Cat# 340542) were purchased from BD Biosciences. MCL-1 (LVUBKM)-PE (Cat# 12-9047-42) was purchased from Invitrogen. TCR Vδ2 (B6)-PE (Cat# 331408) or -APC (Cat# 331418), EGFR (AY13)-APC (Cat# 352906), CD4 (RPA-T4)-FITC (Cat# 300538) or -PE (Cat# 300508), CD8 (SK1)-FITC (Cat# 344704) or -PE (Cat# 344706), CD34 (581)-FITC (Cat# 343504) or -PE (Cat# 343506), CD33 (WM53)-APC (Cat# 303408) or –PE/Cyanine5 (Cat# 303406), CD62L (DREG-56)-

PE/Cyanine7 (Cat# 304822), CD45RA (HI100)-FITC (Cat# 304148), CD45 (HI30)-FITC (Cat# 304006), NKG2D (1D11)-PE (Cat# 320806), DNAM-1 (11A8)-APC (Cat# 338312), CD25 (BC96)-FITC (Cat# 302604), CD69 (FN50)-PE (Cat# 310906), LAG3 (11C3C65)-FITC (Cat# 369308), TIGIT (A15153G)-APC (Cat# 372706), TIM3 (F38-2E2)-PE (Cat# 345006), CD45RA (HI100)-APC (Cat# 304112), CD62L (DREG-56)-Brilliant Violet 650 (Cat# 304832), CCR7 (G043H7)-PE/Cyanine7 (Cat# 353226), BCL-2 (100)-PE (Cat# 658708), Ki67-PE (Cat# 350504), Annexin V-FITC (Cat# 640906) or -Pacific Blue (Cat# 640918) and 7-AAD (Cat# 420404) were purchased from BioLegend. BCL-XL (54H6)-PE (Cat# 13835) was purchased from Cell Signaling Technology. FACS data were analyzed using FlowJo software.

RNA sequencing (RNA-seq) and data analysis

For Figure 3A–B, γδ T cells were drug-treated (venetoclax, 1 μM, 14–18 h). Total RNA of γδ T and ven-γδ T cells from three donors was isolated using TRIzol (Invitrogen, Cat# 15596018). mRNA enrichment was performed on total RNA using oligo(dT)-attached magnetic beads. The enriched mRNA with poly(A) tails was fragmented using a fragmentation buffer, followed by reverse transcription using random N6 primers to synthesize cDNA double strands. The synthesized double stranded DNA was then end-repaired and 5'-phosphorylated, with a protruding 'A' at the 3' end forming a blunt end, followed by ligation of a bubble-shaped adapter with a protruding 'T' at the 3' end. The ligation products were PCR amplified using specific primers. The PCR products were denatured to single strands, and then single-stranded circular DNA libraries were generated using a bridged primer. The constructed libraries were quality-checked and sequenced after passing the quality control. Sequencing was performed on DNBSEQ platform with PE150 (read length). Raw reads were processed by fastp (version 0.20.1) to filter out low quality reads and remove

sequencing adapters. The R package DESeq2 (version 1.38.1) was used to perform differential expression analysis. The significant differentially expressed genes (DEGs) were indicated by fold change > 2 and P value < 0.05, GO, KEGG and GSEA analysis were carried out to explore the function and pathway. Raw and processed RNA-seq data are publicly available in GEO under accession number GSE306856.

Enzyme-Linked Immunosorbent Assay (ELISA)

For cytokine measurements, culture supernatants were clarified (1,000 × g, 5 min, 4°C) and analyzed by ELISA (Cat# KHC4021, Thermo Fisher) according to the manufacturer's instructions. Briefly, 96-well plates pre-coated with capture antibody were brought to room temperature and washed 3 times with 1× wash buffer. Standards were prepared by serial dilution to generate a standard curve in duplicate. Samples were thawed on ice. One hundred microliters of standards, controls, and samples were added to the appropriate wells and incubated for 2 h at room temperature. After 3 washes, 100 μL of HRP-conjugated detection antibody was added to each well and incubated for 1 h at room temperature. Plates were washed again 3 times, and 100 μL of TMB substrate solution was added. Color was allowed to develop for 10–15 min protected from light, and the reaction was stopped with 50 μL of 2 N H_2SO_4. Absorbance was read at 450 nm (reference 570 nm) using a microplate reader. Cytokine concentrations were calculated from the standard curve.

Cell viability

For assessing viability of γδ T cells after venetoclax treatment, cells were seeded into 96-well plates (10,000 cells/100 μL/well) and treated with venetoclax for 14 h. Then the culture media were discarded and replaced by 100 μL of PBS containing 0.1 μL calcein AM (2 mM, Beyotime, Cat# C2012). After incubation, calcein

fluorescence was measured using a microplate reader (excitation, 490 nm; emission, 515 nm). After staining with Hoechst 33342 for 15 min, images were obtained using fluorescence microscope. Moreover, BeyoClick™ EdU Cell Proliferation Kit with Alexa Fluor 488 (Beyotime, Cat# C0071S) was used to assess the vitality of γδ T cells according to manufacturer's instructions.

Seahorse analysis

Oxygen consumption rates (OCR) and extracellular acidification rates (ECAR) of γδ T cells or ven-γδ T cells that had been co-cultured overnight with KG-1α and isolated by positive selection with anti-TCRγδ microbeads (Cat# 130-050-701, Miltenyi Biotec) were measured using Seahorse XFe96 extracellular flux analyzer. These assays were performed according to the manufacturer's instructions with a Seahorse XF Cell Mito Stress Test Kit (Agilent, Cat# 103015-100) and Seahorse XF Glycolysis Stress Test Kit (Agilent, Cat# 103020-100). Briefly, the Seahorse XFe96 FluxPak cartridge was hydrated with distilled water overnight at 37°C (no CO_2) and equilibrated for 1 hour on the day of the assay in Seahorse XF Calibrant solution. Plates were pre-coated with 22.4 μg/mL of Cell-tak (Corning, Cat# 354240) overnight at 4°C to ensure continued adhesion of γδ T cells on the plate during the assay. For OCR testing, cells were cultured in XF DMEM medium supplemented with 10 mM glucose, 1 mM sodium pyruvate, and 2 mM L-glutamine, with pH adjusted to 7.4. 1 μM oligomycin, 2 μM Carbonyl Cyanide 4-(Trifluoromethoxy) Phenylhydrazone (FCCP), and 1 μM rotenone/antimycin A were loaded at the indicated times. To test ECAR, cells were cultured in XF DMEM medium supplemented with 2 mM L-glutamine, with pH adjusted to 7.4. 10 mM glucose, 1 μM oligomycin and 50 mM 2-DG were loaded at the indicated times. Seahorse data were collected using Wave Controller 2.6 (Agilent).

Mitochondrial DNA isolation and real-time polymerase chain reaction

Genomic DNA was extracted using the HiPure Tissue DNA Mini Kit (MAGEN, Cat# D3121-02). Primers for cytochrome c oxidase subunit I (CO1), a key gene for mitochondrial (mt) function and β-actin (Sangon Biotech) were as follows: MT-CO1 (forward, 5'-ATACTACTAACAGACCGCAACCTC-3'; reverse, 5'-GAGATTATTCCGAAGCCTGGT-3'). β-actin (forward, 5'- GAAGGATTCCTATGTGGGCGA-3'; reverse, 5'-CAGGGTGAGGATGCCTCTCTT-3'). Quantitative PCR (qPCR) was performed according to PowerUp™ SYBR™ Green protocols (Thermo Fisher Scientific, Cat# A25741) on an Applied Biosystems QuantStudio 5 sequence detection system. The expression of MT-CO1 was normalized to β-actin.

Mitochondrial mass measurement

Mitochondrial mass was measured using MitoTracker Green (Invitrogen, Cat# M7514) by flow cytometer or fluorescence microscope. For flow cytometry, 2×10^5 γδ T and ven-γδ T cells were stained with 200 nM MitoTracker Green at 37°C for 30 min. For mitochondrial imaging, after MitoTracker Green staining, cells were counterstained with DAPI to visualize nuclei. Images were acquired using a Leica DMi8 inverted microscope.

Metabolite extraction and untargeted metabolomics analysis

For sample preparation, γδ T and ven-γδ T cells from five donors were washed twice with PBS and pelleted at 400 × g for 5 min. Intracellular metabolites were extracted by adding 2 mL ice-cold 80% methanol to the cell pellet, resuspending, and incubating for 2 h at -80°C to precipitate proteins. The methanol extracts were centrifuged at 14,000 × g for 20 min at 4°C, and supernatants were transferred to new tubes on dry ice. Samples were evaporated in a vacuum concentrator for hydrophilic interaction liquid chromatography–

mass spectrometry (HILIC-LC-MS) analysis. Pooled quality control samples were prepared and tested in the same manner as the analytic samples to monitor the stability of the analysis.

LC-MS analysis was performed by Metabolism and lipidomics platform, Tsinghua University (Beijing, China). Briefly, in positive mode, a BEH Amide column (2.1×100 mm, 1.7 um, Waters, USA) was used for separation with column temperature at 40°C. Separation is initiated at 1% mobile phase B with flow rate at 300 μL/min. The gradient is shown as follows: 0 min, 1% B; 2 min, 1% B; 3 min, 5% B; 11 min, 60% B; 14 min, 60% B; 15 min, 99% B; 17 min, 99% B; 17.1 min, 1% B; 20 min, 1% B. In negative mode, BEH C18 column (2.1 × 100 mm ,1.7 um, Waters, USA) was used for separation with column temperature at 40°C. Gradient starts at 1% mobile phase B with flow rate at 220 μL/min shown as follows: 0 min, 1% B; 2 min, 1% B; 12 min, 98% B; 17 min, 98% B; 17.1 min, 1% B; 20 min, 1% B.

For untargeted metabolites screening, Orbitrap Exploris 240 mass spectrometer (Thermo Fisher Scientific, CA) was used. In positive mode, mobile phase A is prepared by dissolving 0.63 g of ammonium formate in 50 mL of HPLC-grade water, then adding 950 mL of HPLC-grade acetonitrile and 1 μL of formic acid. Mobile phase B is prepared by dissolving 0.63 g of ammonium formate in 500 mL of HPLC-grade water, followed by 500 mL of HPLC-grade acetonitrile and 1 mL formic acid. In negative mode, mobile phase A was made by mixing 1 L of HPLC-grade water containing 0.3953 g of ammonium bicarbonate (pH ~8). Mobile phase B contained 80% methanol & 20% acetonitrile.

Tracefinder 3.2 (Thermo, CA) was used for metabolite identification with in-house library containing MS/MS spectra from over 1,500 endogenous metabolites. Mass tolerance of 10 ppm and 15 ppm were

applied for precursor and fragment search. Metabolites were assigned based on fragment matching with MS/MS spectra in the library. Two levels of identification were used: one with MS/MS confirmation and one based only on accurate precursor masses. Chromatographic peak area was used for relative quantitation. RT shift of 0.25 min was allowed for peak alignment. Statistical analysis of differential metabolites analysis and biochemical pathways through metabolic enrichment were performed using MetaboAnalyst 6.0. The complete metabolite concentration matrix from the non-targeted metabolomics profiling is provided in the Data Supplement (Supplemental Table 6).

RNA isolation and qPCR

RNA was isolated using an RNA Rapid Extraction kit (SparkJade, Cat# AC0201) according to the manufacturer's protocol. Complementary DNA was synthesized using RNA to cDNA kit (Takara, Cat# RR037) according to the manufacturer's instructions. Quantitative polymerase chain reaction (qPCR) was performed using SYBR Green supermix (Thermo Fisher, Cat# A25742) on an ABI Prism 7900HT sequence detection system (Applied Biosystems). Results were analysed using SDS 2.4 software (Applied Biosystems). Primer sequences are listed in Supplemental Table 8.

Immunoblotting

γδ T and ven-γδ T cells were lysed in RIPA buffer on ice for 30 min (Beyotime). Lysates were centrifuged at 12,000 × g for 10 min, and supernatants were collected. Samples were separated on precast 4–20% bis-tris protein gels (Epizyme). Separated proteins were then transferred to PVDF membranes and blocked in 5% w/v milk overnight at 4°C. Membranes were then incubated with primary antibodies in 5% w/v BSA in TBS containing 0.1% Tween-20 for 2 h at room temperature, then with HRP-conjugated secondary

antibodies. Antibodies: CPT1A (D3B3) Rabbit Monoclonal Antibody (1:1000, Cat# 12252) and β-Actin (D6A8) Rabbit Monoclonal Antibody (1:1000, Cat# 8457) were all Cell Signaling Technology. HRP-Goat Anti-Rabbit Recombinant Secondary Antibody (H+L) (1:10000, Cat# RGAR001) was purchased from Proteintech. Chemiluminescence detection was used to obtain protein bands.

Confocal imaging

We used MitoTracker Green (Beyotime, Cat# C1048) to stain mitochondria, anti-human TCR Vδ2 antibody (BV421, BioLegend, Cat# 331414) to stain cell membrane and DRAQ5 (Thermo Fisher, Cat# 62254) to stain nuclei. Then live γδ T and ven-γδ T cells were imaged on a confocal laser-scanning microscope (Nikon AX/AXT with nsparc inverted microscope). Images were captured with a 40×/1.2-NA water objective. Imaris Viewer 9.5.1 software was used to process the images.

FAO pathway inhibition with etomoxir

For experiments assessing the contribution of fatty acid oxidation, etomoxir was added to γδ T-cell cultures at a final concentration of 5 μM for 18 h before OCR or cytotoxicity assays. Control cultures received matched vehicle treatment.

178 **Supplemental Figure 1**

179

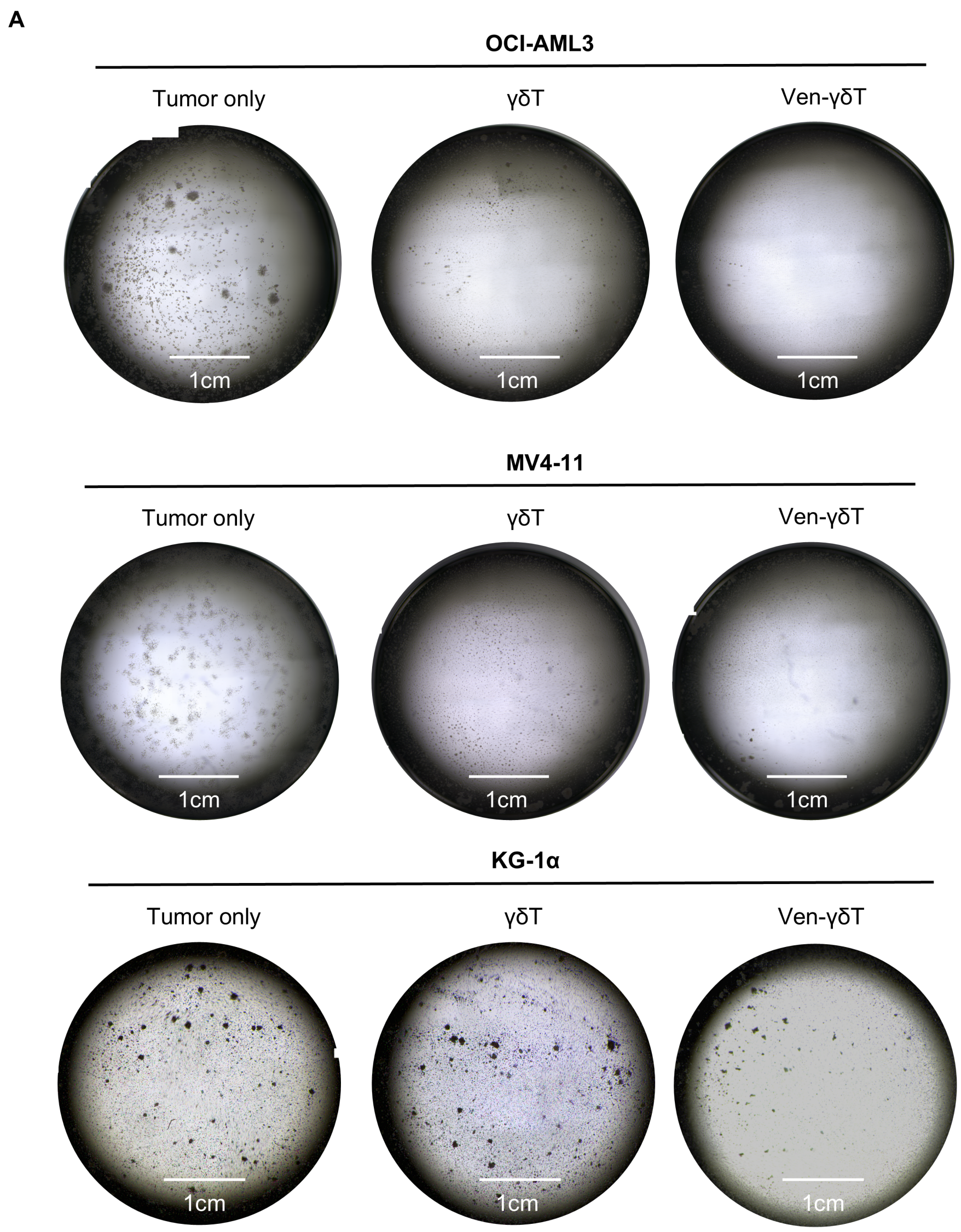

180

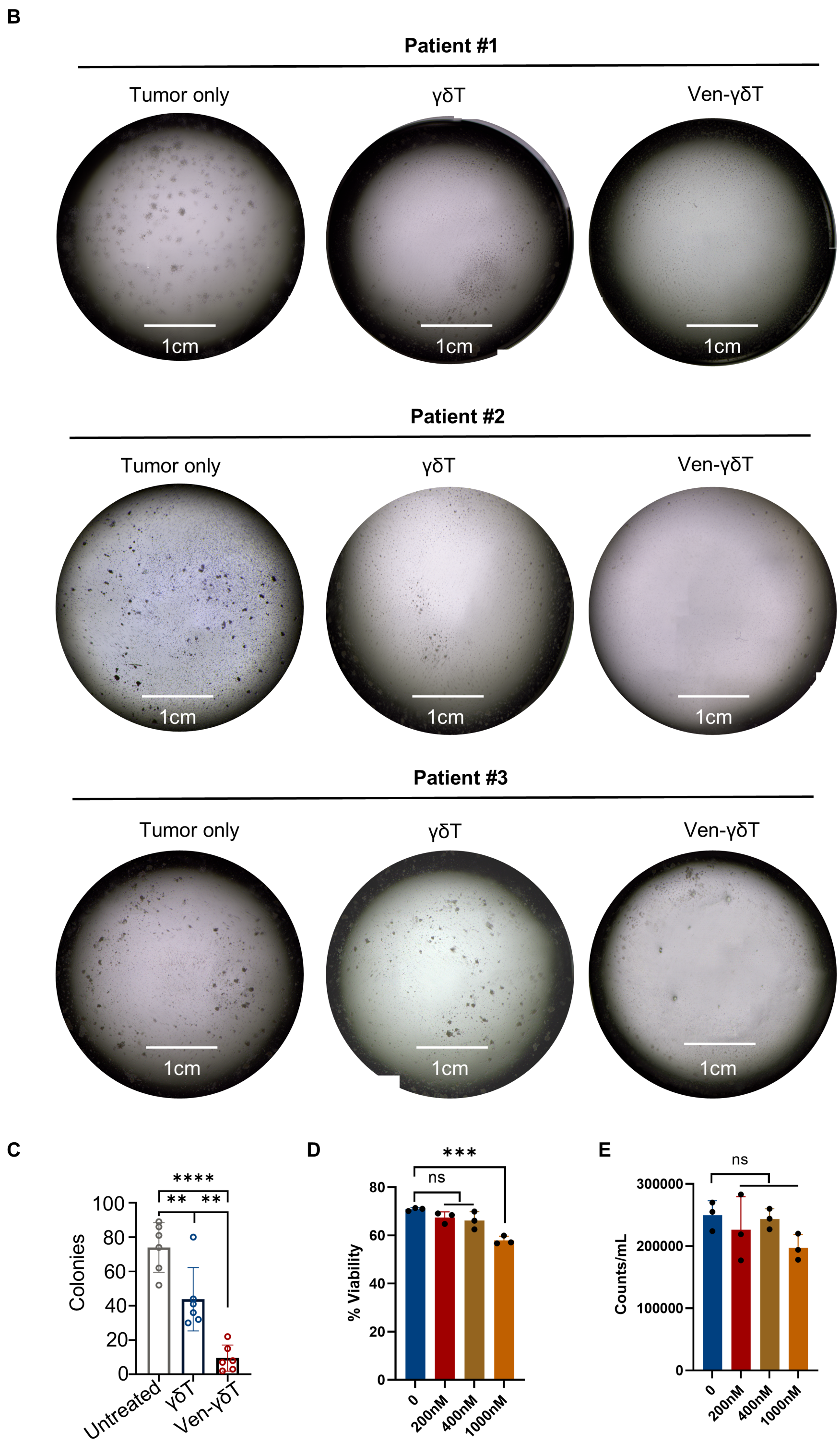

Supplemental Figure 1. Venetoclax enhances γδ T cell-mediated killing of AML cells.

(A-B) Colony formation assay (CFU) assessing the survival of AML cell lines (OCI-AML3, MV4-11, KG-1α) **(A)** and primary AML patient cells **(B)** following co-culture with ven-γδ T cells at a 1:1 E:T ratio for 12–14 h. AML cells (2×10^4 cells per dish) were then seeded and colonies were counted after 14 days.

(C) Colonies were counted after culture in methylcellulose medium, and total CFU numbers are shown. Data are presented as mean ± standard deviation (SD). Data were analyzed using one-way ANOVA with Tukey's comparison test. **, $P < 0.01$; ***, $P < 0.001$; ****, $P < 0.0001$.

(D-E) Viability and cell counts of patient-derived γδ T cells after venetoclax treatment. γδ T cells isolated from patient PBMCs were treated with DMSO or venetoclax at the indicated concentrations (200 nM, 400 nM, and 1000 nM) for 18 h. Cell viability was measured by AO/PI staining (D), and cell numbers were quantified by direct counting (E). Data are presented as mean ± SD. Data were analyzed using repeated-measures one-way ANOVA with Dunnett's multiple-comparisons test. ***, $P < 0.001$.

Supplemental Figure 2

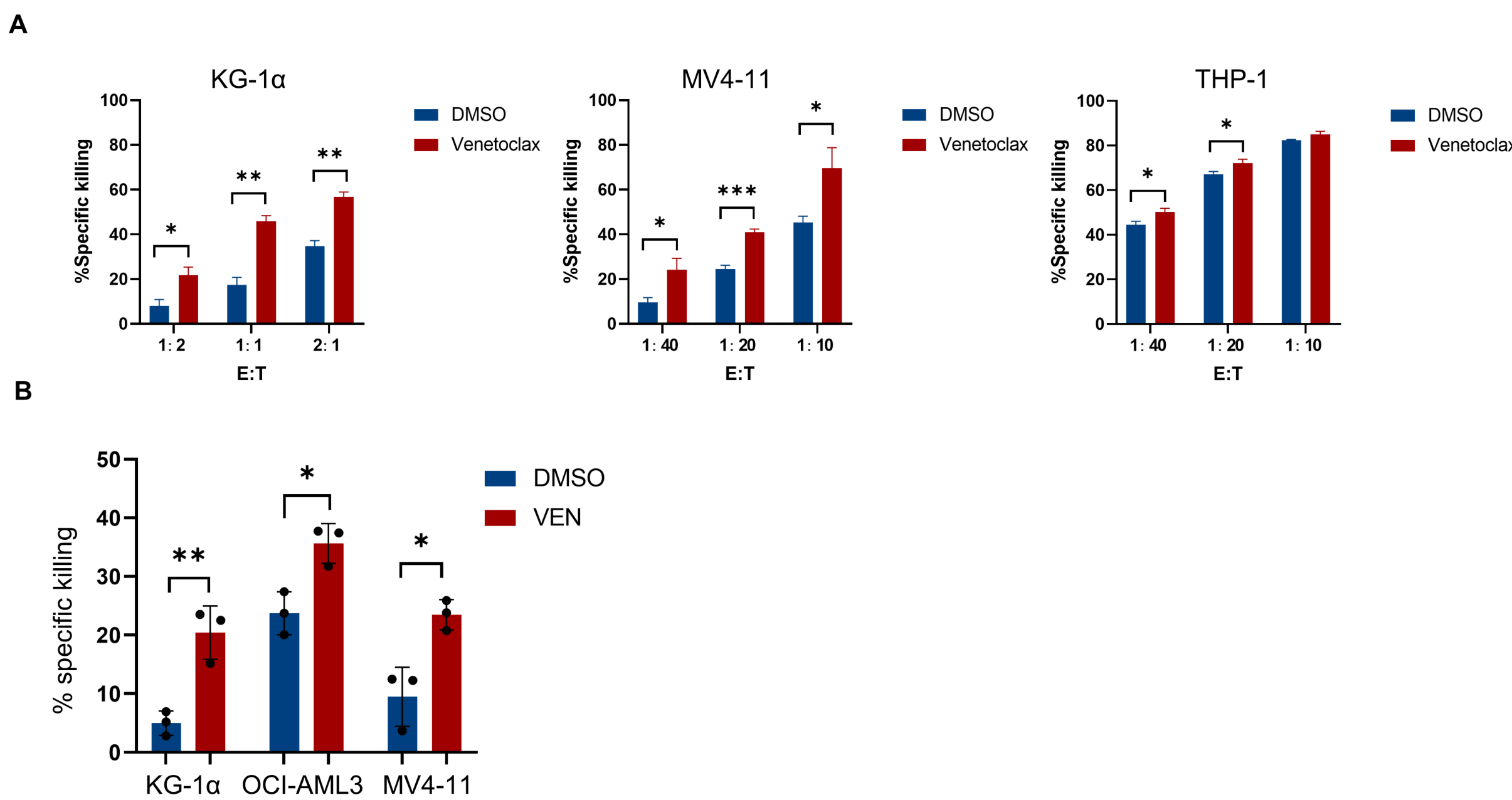

Supplemental Figure 2. Venetoclax enhances the antileukemic activity of Vδ1 and preserves the cytotoxicity of venetoclax-expanded γδ T cells after cryopreservation against AML cell lines.

(A) Vδ1 T cells were obtained from UnicetBio, thawed as described in the Methods, and pretreated *ex vivo* with 1 μM venetoclax or DMSO control before co-culture with AML cell lines. Cytotoxicity against KG-1α, MV4-11, and THP-1 cells was evaluated at the indicated effector-to-target (E:T) ratios.

(B) Cryopreserved γδ T cells previously expanded under venetoclax-conditioned culture were thawed and directly used for co-culture assays to evaluate cytotoxicity against the indicated AML cell lines after recovery.

Data are presented as mean ± standard deviation (SD) and analyzed using two-tailed paired Student's t test. *, $P < 0.05$; **, $P < 0.01$; ***, $P < 0.001$; ****, $P < 0.0001$.

Supplemental Figure 3

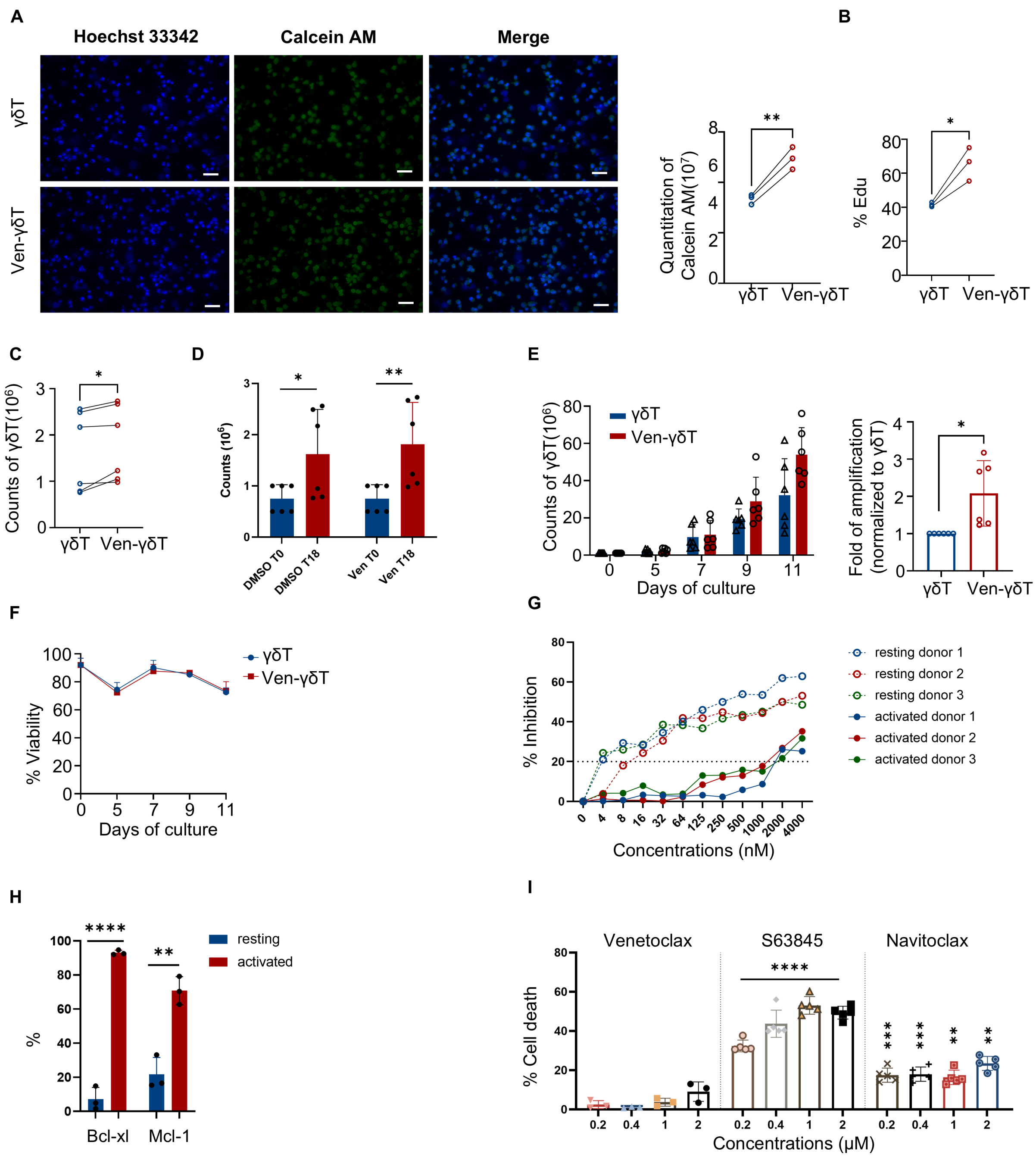

Supplemental Figure 3. Venetoclax enhances proliferation of γδ T cells without affecting their viability.

(A) Immunofluorescence staining of γδ T cells using Calcein AM and Hoechst 33342, with corresponding statistical plots of fluorescence intensity measured by microplate reader. Each point represents an independent donor (n = 3).

(B) Quantification of EdU and Hoechst 33342 staining using flow cytometry. Each point represents an independent donor (n = 3).

(C) Counts of γδ T cells measured after 18-hour treatment with venetoclax (1 μM) or DMSO control under matched culture conditions. Each paired symbol represents γδ T cells from an individual donor (n = 6).

(D) Absolute γδ T-cell counts were measured before treatment and after 18 h of DMSO or venetoclax exposure under matched culture conditions (n = 6).

(E) Fold expansion of γδ T cells cultured with venetoclax (400 nM) over an extended period. Each point represents an independent donor culture (n = 6).

(F) Viability of γδ T cells expanded with or without venetoclax assessed by Acridine Orange (AO) and Propidium Iodide (PI) staining (n = 3).

(G-I) Activated γδ T cells exhibit reduced sensitivity to venetoclax-induced cell death and increased expression of anti-apoptotic proteins. Resting γδ T cells were obtained directly from healthy donor PBMCs, whereas activated cells were γδ T cells stimulated with zoledronic acid. In all flow cytometric analyses, cells were first gated on the γδ T-cell population, and apoptosis or protein expression was then evaluated within the gated population. **(G)** Resting and activated γδ T cells from three independent donors (n = 3) were treated with venetoclax at the indicated concentrations (4–4000 nM), and cell death induction was analyzed by flow cytometry. **(H)** Expression of the anti-apoptotic proteins BCL-XL and MCL-1 in resting

and activated γδ T cells assessed by flow cytometry. **(I)** Activated γδ T cells were treated with venetoclax, the MCL-1 inhibitor S63845, or BCL-2/BCL-XL inhibitor navitoclax at the indicated concentrations, and cell death was determined by flow cytometry.

Data are presented as mean ± standard deviation (SD). Data were analyzed using two-tailed paired Student's *t* test (A-E). Data were analyzed using two-tailed unpaired Student's *t* test (G-I). *, $P < 0.05$; **, $P < 0.01$; ***, $P < 0.001$; ****, $P < 0.0001$.

Supplemental Figure 4

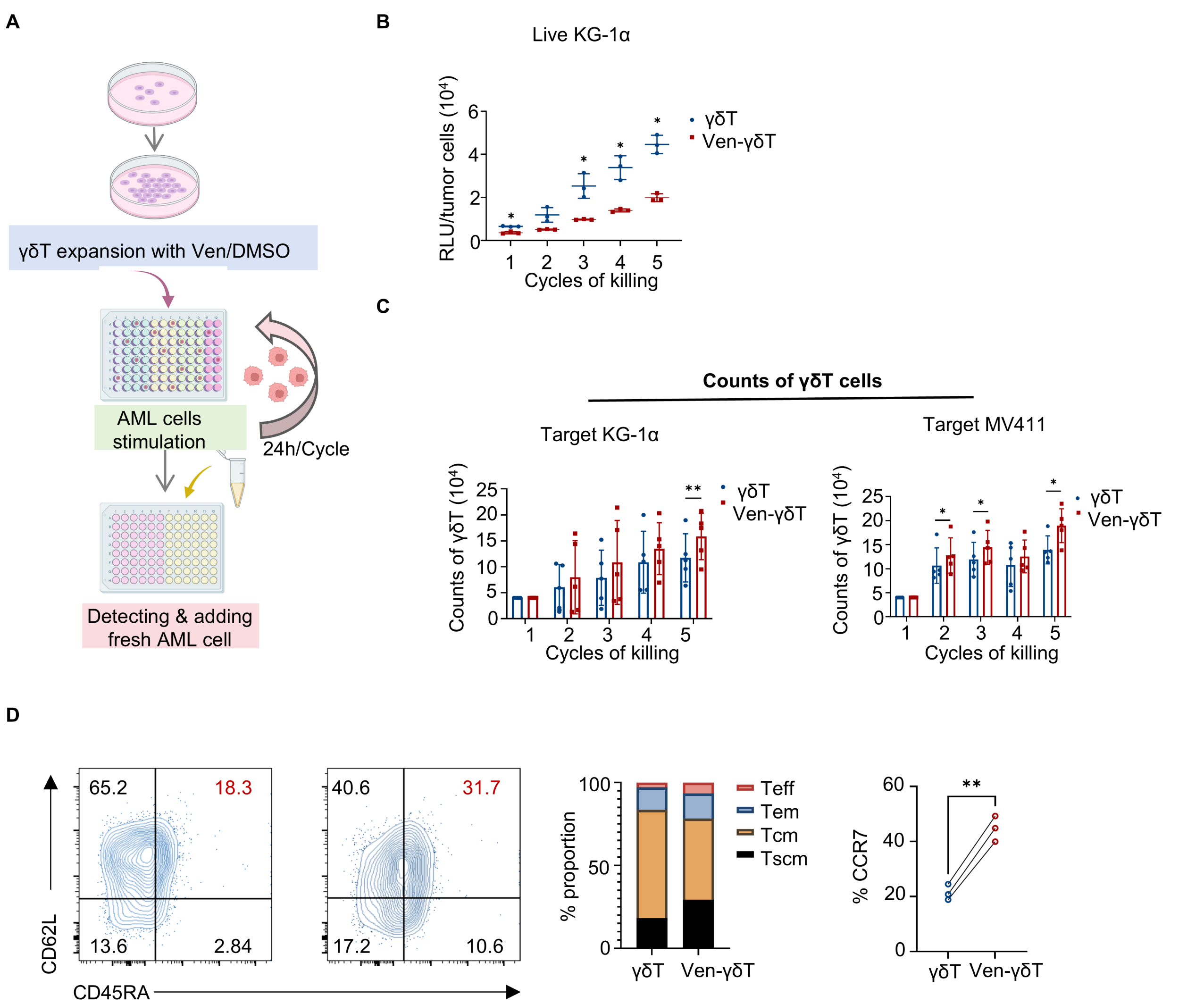

Supplemental Figure 4. Venetoclax promotes long-term cytotoxic activity and memory phenotypic changes of γδ T cells.

(A) Serial killing assay workflow chart.

(B) γδ T cells were expanded with DMSO or venetoclax (400 nM). Then γδ T cells or ven-γδ T cells were stimulated with KG-1α-luc, MV4-11-luc cells at initial E:T ratio of 4:1 at 24-h intervals. AML viability

was determined by luminescence. Data are mean ± SD from triplicate wells. Representative results of n = 3 donors.

(C) Absolute counts of γδ T cells were quantified every cycle. Data are mean ± SD from triplicate wells. Representative results of n = 5 donors.

(D) Flow cytometric analysis and quantification of $CD45RA^{+}CD62L^{+}$ γδ T cells (left), $CCR7^{+}$ γδ T cells (middle), and proportion of T cell memory subsets (right) after venetoclax treatment.

Data are presented as mean ± standard deviation (SD). Data were analyzed using two-tailed paired Student's *t* test. *, P < 0.05; **, P < 0.01; ***, P < 0.001; ****, P < 0.0001.

Supplemental Figure 5

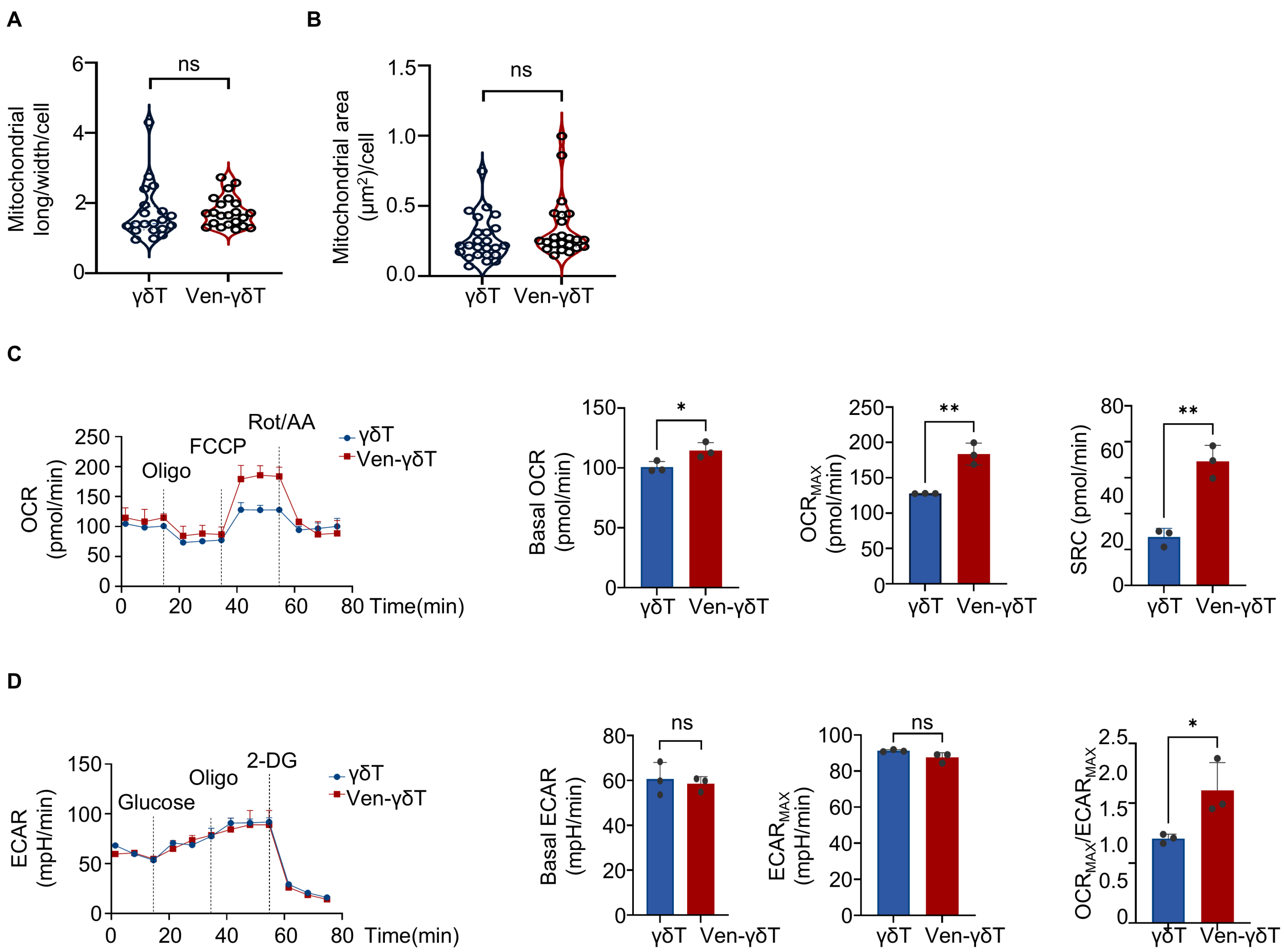

Supplemental Figure 5. Venetoclax promotes oxidative metabolism in γδ T cells.

(A-B) Quantification of length-to-width ratio (A), and area (B) in DMSO- and ven-γδ T cells of transmission electron microscopic (TEM). Data are mean ± SD of over 5 randomly chosen cells per group (n = 3 donors).

(C) Oxygen consumption rate (OCR) traces for DMSO- and ven-γδ T cells with quantification of basal, maximal respiration and spare respiratory capacity (SRC). Data are represented as mean ± SD and are

representative of 3 independent experiments. Oligo, oligomycin; Rot/AA, rotenone and antimycin A; FCCP, Carbonyl Cyanide 4-(Trifluoromethoxy) Phenylhydrazone.

(D) Extracellular acidification rate (ECAR) traces with basal and maximal ECAR quantification. Basal and maximal ECAR values are shown on the right. 2-DG, 2-deoxy-D-glucose.

Data are presented as mean ± SD and analyzed using two-tailed paired Student's t test. *, $P < 0.05$; **, $P < 0.01$; ***, $P < 0.001$.

References

1. Wang Y, et al. B7H3-targeting chimeric antigen receptor modification enhances antitumor effect of Vγ9Vδ2 T cells in glioblastoma. J Transl Med. 2023;21(1):672. doi: 10.1186/s12967-023-04514-8.
2. Zhao X, et al. Targeting C-type lectin-like molecule-1 for antibody-mediated immunotherapy in acute myeloid leukemia. Haematologica. 2010;95(1):71-8. doi: 10.3324/haematol.2009.009811.
