# Supplementary material for: Ex vivo pretreatment with venetoclax boosts antileukemic efficacy of therapeutic γδ T cells in AML models
Source: J Clin Invest. 2026 Jul 23;136(18):e202932. doi: 10.1172/JCI202932 (PMC13574141; doi:10.1172/JCI202932)

Full unedited blot/gel for Figure 6H.

CPT1A

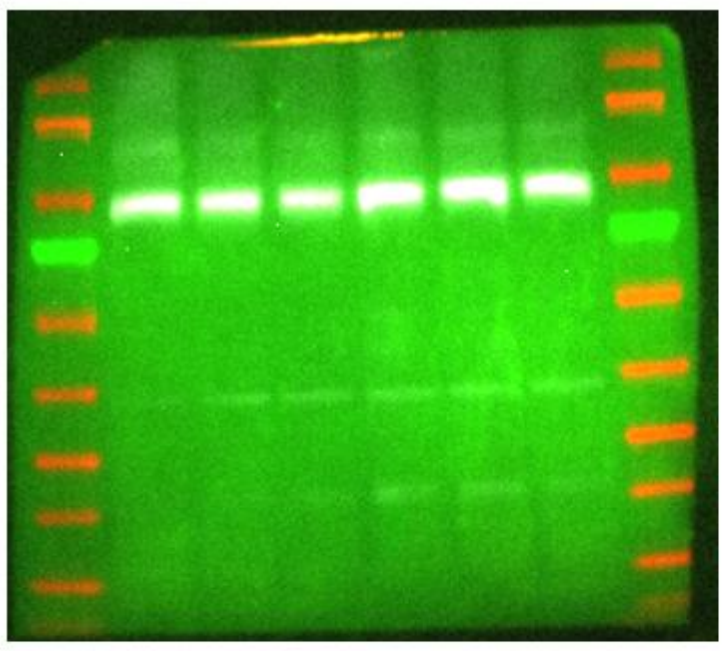

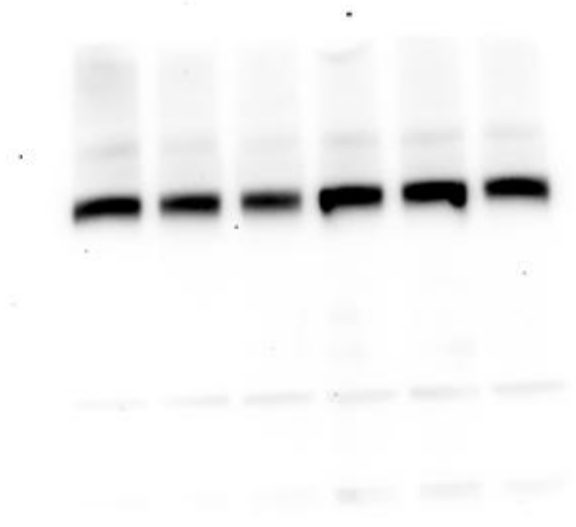

β-actin

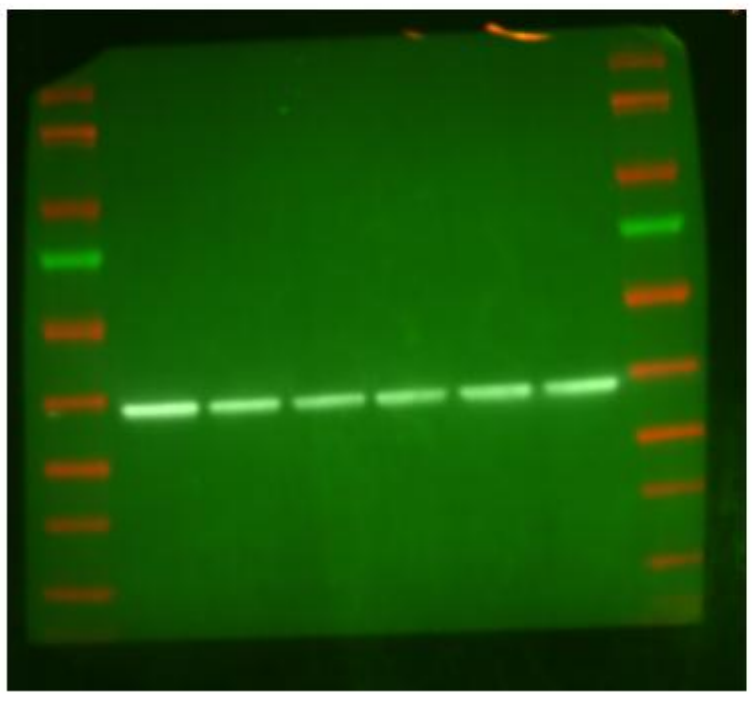

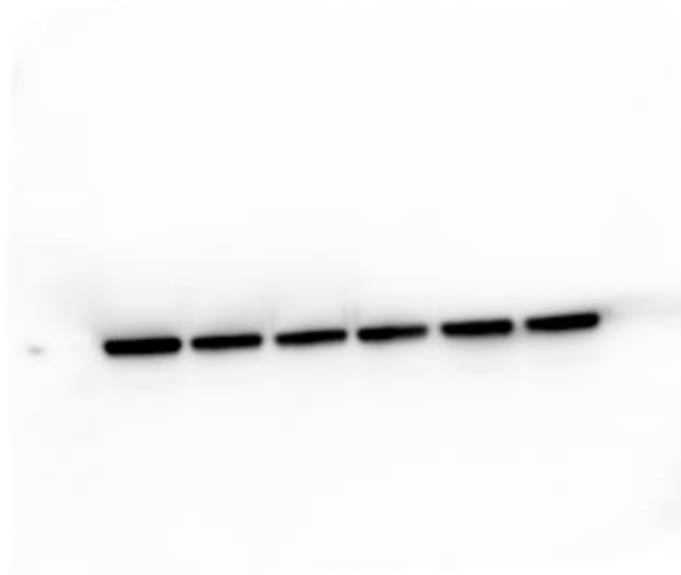

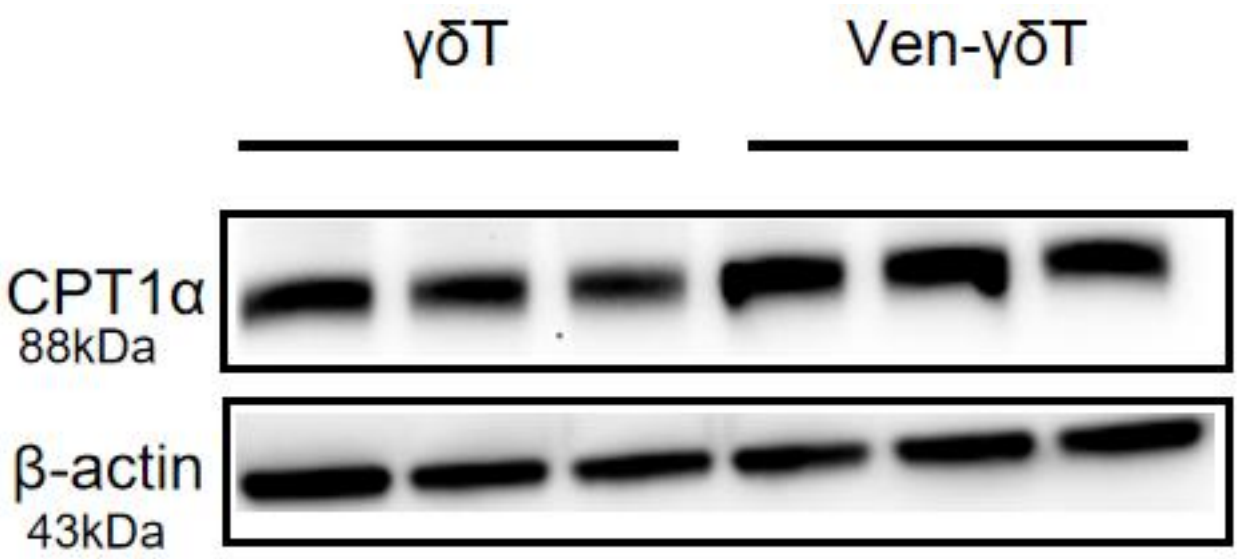

Supplement: Supplementary file 2 [file jci-136-202932-s154.pdf]
